# A Micropatterned Human‐Specific Neuroepithelial Tissue for Modeling Gene and Drug‐Induced Neurodevelopmental Defects

**DOI:** 10.1002/advs.202001100

**Published:** 2021-01-06

**Authors:** Geetika Sahni, Shu‐Yung Chang, Jeremy Teo Choon Meng, Jerome Zu Yao Tan, Jean Jacques Clement Fatien, Carine Bonnard, Kagistia Hana Utami, Puck Wee Chan, Thong Teck Tan, Umut Altunoglu, Hülya Kayserili, Mahmoud Pouladi, Bruno Reversade, Yi‐Chin Toh

**Affiliations:** ^1^ Department of Biomedical Engineering National University of Singapore Singapore 117583 Singapore; ^2^ NUS Tissue Engineering Program National University of Singapore Singapore 119077 Singapore; ^3^ Institute for Health Innovation & Technology (iHealthTech) National University of Singapore Singapore 117599 Singapore; ^4^ Divison of Engineering New York University Abu Dhabi 129188 United Arab Emirates; ^5^ Department of Mechanical Engineering New York University New York NY 11201 USA; ^6^ Institute of Medical Biology Human Genetics and Embryology Laboratory A*STAR Singapore 138648 Singapore; ^7^ Translational Laboratory in Genetic Medicine (TLGM) Agency for Science, Technology, and Research (A*STAR) Singapore 138648 Singapore; ^8^ Istanbul Medical Faculty Medical Genetics Department Istanbul 34093 Turkey; ^9^ Koç University School of Medicine Medical Genetics Department Istanbul 34010 Turkey; ^10^ Department of Medicine National University of Singapore Singapore 119228 Singapore; ^11^ Institute of Molecular and Cellular Biology A*STAR Singapore 138673 Singapore; ^12^ Amsterdam Reproduction and Development Academic Medical Centre and VU University Medical Center Amsterdam 1105 the Netherlands; ^13^ National University of Singapore Department of Pediatrics Singapore 119228 Singapore; ^14^ The N.1 Institute for Health National University of Singapore Singapore 117456 Singapore; ^15^ School of Mechanical Medical and Process Engineering Queensland University of Technology Brisbane Queensland 4000 Australia; ^16^ Institute of Health and Biomedical Innovation Queensland University of Technology Kelvin Grove Queensland 4059 Australia

**Keywords:** human pluripotent stem cells, micropatterning, morphogenesis, neurodevelopmental defects, neuroepithelium

## Abstract

The generation of structurally standardized human pluripotent stem cell (hPSC)‐derived neural embryonic tissues has the potential to model genetic and environmental mediators of early neurodevelopmental defects. Current neural patterning systems have so far focused on directing cell fate specification spatio‐temporally but not morphogenetic processes. Here, the formation of a structurally reproducible and highly‐organized neuroepithelium (NE) tissue is directed from hPSCs, which recapitulates morphogenetic cellular processes relevant to early neurulation. These include having a continuous, polarized epithelium and a distinct invagination‐like folding, where primitive ectodermal cells undergo E‐to‐N‐cadherin switching and apical constriction as they acquire a NE fate. This is accomplished by spatio‐temporal patterning of the mesoendoderm, which guides the development and self‐organization of the adjacent primitive ectoderm into the NE. It is uncovered that TGF*β* signaling emanating from endodermal cells support tissue folding of the prospective NE. Evaluation of NE tissue structural dysmorphia, which is uniquely achievable in the model, enables the detection of apical constriction and cell adhesion dysfunctions in patient‐derived hPSCs as well as differentiating between different classes of neural tube defect‐inducing drugs.

## Graphical abstract

This paper reports the generation of a reproducible and highly organized neuroepithelial (NE) tissue by combining human pluripotent stem cell (hPSC) micropatterning and a temporally sequenced induction protocol to specify NE cells in spatial juxtaposition to mesoendoderm cells. By evaluating NE tissue structural dysmorphia in the micropatterned NE model, we can successfully model gene‐ and drug‐induced neurodevelopmental defects.

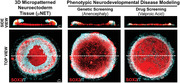

## 1 Introduction

Human pluripotent stem cells (hPSCs) are increasingly being used as in vitro models to mimic human embryonic development because they can recapitulate both cellular differentiation and tissue organization (i.e., morphogenesis) processes.^[^ ^1^ ^]^ Current hPSC‐based models have so far relied on measuring molecular markers and metabolites to detect disruption in cellular differentiation processes^[^ ^2^, ^3^, ^4^, ^5^, ^6^, ^7^ ^]^ because conventional differentiation protocols yield structurally heterogeneous tissues, which cannot provide a consistent baseline to track tissue organization. Recently, micropatterned hPSCs have been used to generate structurally‐standardized embryonic tissues by confining differentiating stem cells in predefined geometries to establish spatial patterning of mechanical and soluble signaling cues.^[^ ^8^, ^9^, ^10^, ^11^, ^12^ ^]^ This has catapulted the idea of measuring morphological changes in micropatterned hPSC‐derived embryonic tissues to screen and identify potential molecular mediators involved in developmental defects, similar to how structural abnormalities are used as a readout in developmental organisms to indicate for birth defects.^[^ ^13^, ^14^ ^]^ To date, this concept has been demonstrated in a number of micropatterned tissues, including amniotic,^[^ ^15^ ^]^ mesodermal,^[^ ^16^, ^17^ ^]^ and cardiac^[^ ^18^ ^]^ tissues. However, morphological phenotyping of a micropatterned hPSC‐derived neural tissue to model and screen genetic and environmental factors involved in early neurodevelopment dysfunctions has not been demonstrated.

Early neurodevelopment is initiated by neurulation, which results in the formation of the neural tube. This key developmental transition is a multi‐step process, characterized by a series of cell differentiation events to generate various ectodermal derivatives (i.e., neuroepithelium, neural crest, sensory placodes, and epidermis),^[^ ^12^ ^]^ which are tightly coordinated with a form of tubulogenesis, characterized by a tissue wrapping mechanism involving actomyosin contractility and planar cell polarity (PCP).^[^ ^19^, ^20^ ^]^ Recent works on neural tissue patterning in vitro have demonstrated that spatio‐temporal control of soluble morphogens can direct the patterning of various ectodermal derivatives.^[^ ^12^, ^21^ ^]^ However, the recapitulation of morphogenetic cellular processes relevant to neurulation remains unexplored.

To this end, we have developed a structurally‐standardized human neuroepithelial (NE) tissue that recapitulates morphogenetic cellular processes relevant to early neural development. While the NE is capable of self‐organizing into two‐dimensional (2D)/three‐dimensional (3D) neural rosettes in vitro,^[^ ^21^, ^22^ ^]^ early works in developmental biology have demonstrated that the morphogenetic organization of the developing NE tissue does not occur in isolation but requires interplay with surrounding embryonic tissues.^[^ ^21^, ^22^ ^]^ This suggests the importance of recapitulating germ layer interactions in controlling NE morphogenesis, akin to epithelial–mesenchymal interactions during in vitro organization of other epithelial tissues^[^ ^23^, ^24^ ^]^ hPSC‐derived mesoendoderm (ME) cells have been recently shown to exhibit primitive streak‐like properties.^[^ ^25^ ^]^ Therefore, we hypothesized that a non‐neural embryonic tissue at a developmentally‐relevant stage, such as the ME, can be used to guide the organization of the epiblast into NE in vitro. Hence, we extended the capability of micropatterned hPSCs in organizing stem cell differentiation fates^[^ ^9^, ^10^, ^11^, ^15^, ^18^ ^]^ to consistently induce and pattern NE cells in spatial juxtaposition to ME cells within a defined tissue geometry. This resulted in a structurally reproducible NE tissue, which allows us to recapitulate morphogenetic processes in the NE, including E‐to‐N cadherin (ECAD‐to‐NCAD) switching and actomyosin contractility. We showed that hPSCs bearing genetic mutations, which have molecular targets involved in these morphogenetic processes, resulted in aberrant NE tissue structures. Morphological assessment of the emergent ME–NE tissue organization could also detect neural tube defect (NTD) inducing drugs. This demonstrated the successful exploitation of NE tissue structure phenotyping for testing and investigating gene and environmental mediators of neurodevelopmental defects.

## 2 Results

### 2.1 Generating Micropatterned Neuroepithelial Tissue with Distinct Tissue Architectures

**Figure 1:**
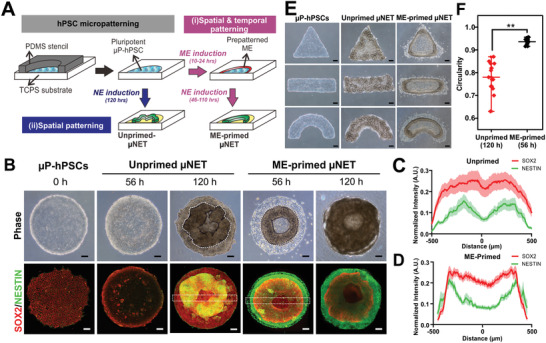
Formation of micropatterned neuroepithelial tissues (μNET) with different architectures. A) Generation of micropatterned hPSC (*μ*P‐hPSCs) followed by different induction processes to derive unprimed and mesoendoderm (ME)‐primed μNET structures, respectively. B) Gross morphologies of unprimed and ME‐primed μNETs at 56 and 120 h post differentiation. Top panel shows phase contrast images. Lower panel shows corresponding immunofluorescence images labeling neuroepithelium markers, SOX2 and NESTIN. C,D) Quantification of expression SOX2 and NESTIN across the micropatterns in (C) unprimed and (D) ME‐primed μNETs (56 h), measured by intensity profile along white dotted cross‐sections in (B). Data are average of ± s.e.m of ≥15 cross‐sections from ≥5 colonies from three independent experiments. E) Unprimed and ME‐primed μNETs generated from micropatterned hPSCs of different geometries with colony area equivalent to 1000 µm diameter circular micropattens. F) Quantification of tissue circularity of ME‐primed and unprimed μNETs, denoted by white dotted lines in (B). Data are average of ± s.e.m of 16 colonies from five independent experiments. **p < 0.00005 (Student's *t*‐test). Scale bars in (B–E) = 100 µm. All μNETs were generated from H9 hESCs.

We spatially directed the formation of two architecturally distinct 3D NE tissues (hereafter referred to as micropatterned‐neuroepithelial tissues, μNET) in micropatterned hPSC colonies, which were subjected to either NE induction alone or ME and NE inductions that were temporally sequenced in a developmentally‐inspired manner (**Figure** **1**A). The latter spatio‐temporal patterning would allow the cells at the colony periphery to be prepatterned into a ME fate,^[^ ^8^ ^]^ while allowing cells in the rest of the colony to acquire a NE fate, thereby allowing us to follow NE development in the presence of spatially‐juxtaposed ME cells.

When hPSC colonies were directly induced into NE within the confinement of a micropattern, we could observe the formation of an irregular SOX2^+^/NESTIN^+^ 3D rosette‐like structure after 120 h (Figure 1B,C). When the micropatterned hPSC colony was pulsed with ME induction medium for 10 h prior to NE induction, it resulted in the formation of a symmetrical SOX2^+^/NESTIN^+^ 3D annular structure within 56 h of differentiation (Figure 1B,D). The duration of ME induction was found to be critical. 10 h of ME induction was the minimum period required to observe the expression of T on the edge of the micropatterns (Figure S1A, Supporting Information), which in turn and generated symmetrical annular structures that could remain adherent for an equivalent amount of time (i.e., 120 h) required to generate the unprimed μNET structures for subsequent comparative studies (Figure S1B, Supporting Information). The observed emergent tissue structures were confirmed to be a result of neural‐specification under geometrical confinement since micropatterned hPSCs maintained in self‐renewal (Figure S1C, Supporting Information) or basal^[^ ^17^ ^]^ medium did not form these structures. The overall geometries of both unprimed and ME‐primed μNETs can generally be dictated by the shape of the hPSC micropatterns. However, the former resulted in geometrical tissue structures that were highly tortuous and variable between different colonies (Figure 1E,F). In contrast, the geometry of the ME‐primed μNETs faithfully conformed to that of the hPSC micropatterns (Figure 1E) with minimal inter‐colony variability of <1.5%, as indicated by the circularity shape factor of 0.94 ± 0.014 (Figure 1F). Therefore, temporally‐sequenced ME and NE induction of micropatterned hPSC colonies was able to generate NE tissues that were architecturally more ordered and consistent than NE induction alone.

### 2.2 Cell Fates Characterization in ME‐Primed and Unprimed μNETs

We first verified for the presence of ME lineages in the ME‐primed μNETs, as indicated by the upregulation of Brachyury (*T*), *Sox17*, *FoxA2* transcripts at 56 h post differentiation, which were absent in the unprimed μNETs (Figure S2, Supporting Information). The transcriptional expressions of pan‐NE markers (*SOX2*, *SIP1*, *NCAM*, *NCAD*) were comparable between the unprimed and ME‐primed μNETs. NE markers were weakly expressed at 56‐h, and robustly expressed only at 120 h, which agreed with previous reports.^[^ ^26^ ^]^ This suggested that at 56 h, ME cell fate specification was completed whereas NE cell fate specification has just been initiated in the μNETs. Interestingly, we could observe differences in the expression levels of region‐specific NE markers along the anterior–posterior neuroaxis between the ME‐primed and unprimed μNETs. As early as 56 h, ME‐primed μNETs had a more posterior‐biased expression of genes such as *GBX2*, *FGF8*, and *PAX5*, while unprimed μNET expressed higher level of anterior genes such as *OTX2* (Figure S2, Supporting Information). In addition, ME‐primed μNET also exhibited an enrichment of *HOXA2*, *HOXA9*, *HOXA11* at 150 h post differentiation (Figure S2, Supporting Information). This suggests that the presence hPSC‐derived ME tissue could induce the neighboring NE to acquire a more posterior identity. The differences in the expression of non‐neural ectodermal (*GATA2*, *DLX3*) and neural plate border cell (*PAX3*, *PAX7*) identities between ME‐primed and unprimed μNET are statistically insignificant, indicating that ME cells alone could not support the development of all ectodermal derivatives. (i.e., non‐neuroectoderm and neural plate border cells).

**Figure 2:**
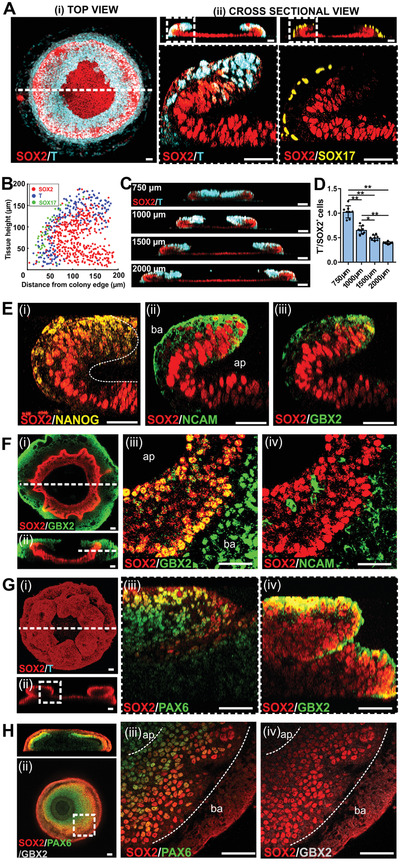
Spatially juxtaposed ME cells altered the 3D cellular organization of resultant NE tissue. A) Immunofluorescence labeling of SOX2^+^, T^+^, and SOX17^+^ cells showing laminar organization in a 56‐h old ME‐primed μNET. (i) Maximum projection of a 3D confocal section of the entire ME‐primed μNET. (ii) Cross‐sectional views of 3D confocal sections of whole ME‐primed μNETs (top panels) and magnified regions (white dotted box) where 3D tissue folding occurred (bottom panels). B) Localization map of SOX2^+^, T^+^ and SOX17^+^ cells along *x*‐*z* cross‐sectional plane of seven independent ME‐primed μNETs, showing a high degree of consistency in the laminar organization of the different cell lineages between different colonies. C) Cross‐sectional views of 3D confocal sections of 56‐h old ME‐primed μNETs generated from hPSC micropatterns with different diameters. D) Quantification of T^+^ to SOX2^+^ cell ratio in ME‐primed μNETs generated from hPSC micropatterns with different diameter. Data are average of ± s.e.m of eight cross sections from four independent samples. (One‐way ANOVA followed by Tukey´s post‐test, **p < 0.0001, *p < 0.005). E) Expression patterns of (i) pluripotent (NANOG) and (ii,iii) neuroepithelium (NCAM and GBX2) markers in 56‐h old ME‐primed μNETs. Images are magnified cross‐sectional views of 3D confocal sections where tissue folding occurred. F) Expression patterns of neuroepithelium markers in 120‐h old ME‐primed μNETs. (i) Maximum projection and (ii) cross‐sectional views of 3D confocal sections of whole ME‐primed μNETs showing that the laminar organization of the NE was preserved. (iii,iv) Magnified view of single optical section transversing the ME‐primed μNETs along the dotted white line in (ii), showing spatial localization of (iii) GBX2 and (iv) NCAM together with SOX2. G) Expression patterns of neuroepithelium markers in 120‐h old unprimed μNETs. (i) Maximum projection and (ii) cross‐sectional views of 3D confocal sections of whole unprimed μNETs labelled with SOX2 and T showing absence of other germ layers in direct NE induction. (iii,iv) Magnified cross‐sectional views of region where 3D tissue protrusion occurred (delineated by white dotted box in (ii)) indicating that the protruded tissue was enriched for (iii) PAX6 and (iv) GBX2. H) Expression patterns of neuroepithelium markers in 120‐h old ME‐primed μNETs generated from anteriorizing NE induction with dual SMAD inhibition. (i) Cross‐sectional side view of whole μNETs and (ii) single optical sections transversing the μNETs along the dotted white line labelled with SOX2, PAX6 and GBX2. (iii,iv) Magnified view of ME‐primed μNETs along the dotted box in (ii), showing spatial localization of (iii) PAX6 and (iv) GBX2 together with SOX2. Scale bars in (A,E–G) = 50 µm, C) = 100 µm, ap—apical, ba—basal. All μNETs were generated from H9 hESCs.

Whole mount immunofluorescence staining was then employed to map out the organization of the different cell lineages in the μNETs at different time points. The 3D annular structure formed in the ME‐primed μNETs at 56 h was observed to be a result of tissue folding of the epithelium at the colony periphery toward the colony center (**Figure** **2**A). A highly reproducible laminar organization could be observed in the 3D tissue fold, with the outer layer being SOX17^+^ and T^+^, which are indicative of the ME; while the inner layer was SOX2^+^/NANOG^−^, which is indicative of a primitive ectoderm that was transiting out of pluripotency and becoming the prospective NE (Figure 2B,E(i)). The ME cells were restricted to the periphery folded regions and were absent in the interior of the colony. The dynamics of tissue folding can be precisely controlled by varying the colony size (Figure 2C,D). A smaller colony resulted in a higher proportion of ME (T^+^) to prospective NE (SOX2^+^) cells (Figure 2D), which would increase the extent of folding (Figure 2C). At this early time‐point, we could only observe modest expression of selected NE markers at the folding region (i.e., NCAM, GBX2) (Figure 2E(ii,iii)). These NE markers were initially colocalized with the ME layer, but upon further maturation to 120 h, their expression region expanded and became segregated from the SOX17^+^ and T^+^ cells (Figure S3A,B, Supporting Information) while still retaining an apico‐basal arrangement with the SOX2^+^ layer (Figure 2F). The expressions of other key NE markers, including PAX6, OTX2 were only observed at 120 h (Figure S3C, Supporting Information), which concurred with the gene expression data (Figure S2, Supporting Information). However, they were mainly localized to the non‐bending regions in the center of the micropatterned colony that lacked juxtaposition with the ME tissue (Figure S3C, Supporting Information). Dorsal neural plate border cells, marked by SOX10 and PAX3, were completely absent in the entire ME‐primed μNET construct (Figure S3C, Supporting Information), which also agreed with the gene expression results. In contrast, the highly tortuous annular structures seen in unprimed μNETs were formed as a result of 3D multi‐cellular protrusions from the micropatterned colony. The 3D protrusions exclusively expressed NE markers, which had a higher proportion of PAX6^+^ cells than GBX2^+^ cells (Figure 2G), confirming a more anterior neural identity (Figure S2, Supporting Information).

Our results collectively indicated that in the ME‐primed model, spatial juxtaposition with ME resulted in the induction of local tissue bending of the adjacent SOX2^+^/NANOG^−^ primitive ectoderm epithelium at 56 h post differentiation that was otherwise absent in the unprimed model. This organization would persist as the primitive ectoderm matures into the NE by 120 h post differentiation. The presence of ME cells also caused the posteriorization of the NE tissue derived from the bending primitive ectoderm at a later time point (120 h) as indicated by the preferential expression of GBX2 over PAX6. The posteriorization of NE by hPSC‐derived ME is in concordance with a recent seminal work by Martyn et al., demonstrating that hPSC‐derived ME cells induces a secondary neural axis that is predominantly posterior (OTX2^−^/GBX2^+^/HOXB1^+^) when transplanted into a chick embryo.^[^ ^25^ ^]^ To demonstrate that early ME‐mediated folding of the prospective NE was decoupled from subsequent NE posteriorization events, ME‐primed μNETs were generated using NE induction medium with dual SMAD inhibition, which has been shown to be highly efficient in driving anterior NE specification.^[^ ^27^, ^28^ ^]^ Indeed, we observed that these ME‐primed μNETs retained their capacity to undergo tissue folding into an organized annular structure (Figure 2H(i,ii)). However, the folded NE tissue now preferentially expressed PAX6 over GBX2 at 120 h (Figure 2H(iii,iv)), which is indicative of a more anterior neural fate. Hence, we surmised that the prepatterned hPSC‐derived ME may mediate local folding and specification of the adjacent primitive ectoderm into a NE that is plastic in its anterior‐posterior identity.

### 2.3 ME‐Primed μNETs Recapitulates Morphogenetic Cellular Processes Involved in Early NE Development

**Figure 3:**
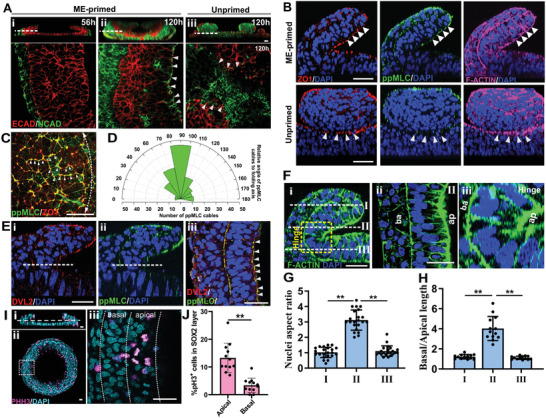
Distinct tissue architectures in ME‐primed and unprimed μNETs were mediated by differences in morphogenetic cellular processes. A) Expression patterns of N‐cadherin (NCAD) and E‐cadherin (ECAD) cell adheren junctions in (i) 56‐h old ME‐primed μNET, (ii) 120‐h old ME‐primed μNET and (iii) 120‐h old unprimed μNET. Images are cross‐sectional views of 3D confocal sections (top panel) and magnified view of single optical sections transversing the μNETs along the dotted white lines (bottom panel). Segregation of ECAD and NCAD are indicated by white arrows. (B) Expression patterns of apical constriction markers ZO1, ppMLC and F‐actin in ME‐primed and unprimed μNETs. Images are magnified cross‐sectional side views of 3D confocal optical sections of μNETs. (C) Merged image of ZO1and ppMLC in ME‐primed μNETs at 34 h, prior to the tissue folding, marking ppMLC cables (arrowheads) along the prospective folding axis (white dotted line). (D) Quantification of angular distribution of ppMLC cables, that linearly extended more than 10 µm across multiple cells, relative to prospective folding axis (white dotted line in (C)). Data are average of ten to 15 cables per image from nine independent samples. E) Expression patterns of planar cell polarity marker, DVL2, and ppMLC in ME‐primed μNETs. Images are (i,ii) cross‐sectioned side view and (iii) magnified view of single optical section transversing at the hinge region in μNETs (along the dotted white lines) displaying apical colocalization of DVL2 and ppMLC. F) F‐actin staining showing prospective NE cell morphologies at (i) magnified cross‐section side view of fold showing different optical sections transversing the ME‐primed μNETs at different *z*‐axis, (ii) single optical section transversing the μNETs along section (II) showing elongated pseudostratified columnar like cells on apical (ap) side of the fold, and (iii) magnified view of hinge displaying wedge‐shaped cells at the fold, with shorter cell length at apical (ap) side and longer cell length on the basal (ba) side of NE tissue. G) Quantification of nuclei elongation as measured by the aspect ratio of individual nuclei for optical section transversing the μNETs at I, II, and III. H) Quantification of cell shape as measured by the ratio of basal to apical cell length for side view sections at I, II(hinge), and III (equivalent area for each section as yellow dotted square in D(i). Data are average of ± s.e.m of 20 nuclei per slice from four independent samples (One‐way ANOVA followed by Tukey´s post‐test, **p < 0.0001). I) Expression patterns of mitotically active phospho‐histone3 (PH3)^+^ cells in 56‐h old ME‐primed μNETs. (i) Cross‐sectional view and (ii) top view of whole μNET structure with μNET; (iii) single optical section showing magnified view of cells at the hinge region, as indicated by white line in (i) tranversing white box region in (ii). J) Quantification of % PH3^+^ cells at the apical and basal side of in SOX2^+^ cell layer in μNETs at the hinge region. Data are average of ± s.e.m of 12 slices from three independent samples (Student's *t*‐test, **p < 0.0001). Scale bars = 50 µm, C, Fii, Eiii = 20 µm.

We then attempted to probe for specific cellular morphogenetic processes involved in the formation of different NE tissue architectures in the presence or absence of lateral ME tissues. Cadherin‐mediated cell adhesions are important in modulating tissue morphogenesis, including neurulation.^[^ ^29^ ^]^ It has been shown that the nascent neural plate in chick embryos is initially positive for ECAD but negative for NCAD, and undergoes progressive cadherin switching to express NCAD by the end of neurulation.^[^ ^30^ ^]^ ECAD and NCAD organization in a 56‐h old ME‐primed μNET closely resembled that of the neural plate in a HH4 chick embryo,^[^ ^30^ ^]^ where ECAD was colocalized with the SOX2^+^ prospective NE layer while NCAD was predominantly expressed in underlying ME tissues (**Figure** **3**A(i)). Upon further maturation to 120 h, NCAD could also be detected at the apical side of the ECAD^+^ NE layer (Figure 3A(ii)), which parallels the expression patterns in HH8‐9 chick embryos, when ECAD‐to‐NCAD switching is being initiated.^[^ ^30^ ^]^ In comparison, the expression patterns of ECAD and NCAD in the unprimed μNETs corresponded to tissue patterns that would emerge from cell sorting processes according to the differential adhesion hypothesis.^[^ ^31^ ^]^ NCAD was localized predominantly in the 3D protruded structure where neural markers were highly expressed; while ECAD was localized to the underlying epithelial layer (Figure 3A(iii)).

The bending of a developing NE is driven by apical constriction, which is denoted by the concentration of the actomyosin contractile machinery to the adheren junctions at the apical cell domain.^[^ ^32^ ^]^ Although diphosphorylated myosin lightchain (ppMLC) was observed at both the interface of the ME‐NE tissue compartments and the apical domain of the prospective NE, its colocalization with Zonula occludens‐1 (ZO‐1) was only found at the apical domain of the prospective NE layer (Figure 3B, Video S1, Supporting Information), which was similarly observed in a folding NE.^[^ ^33^ ^]^ In contrast, there was no polarized localization of ZO‐1 and ppMLC in the unprimed μNETs (Figure 3B). In addition, we also examined the distribution of ppMLC in the apical surface of 34‐h old ME‐μNETs just before tissue folding occurs. It was observed that individual cells displayed ppMLC‐rich and ppMLC‐poor borders, whereby the ppMLC‐rich borders of multiple cells would connect together to form a linear ppMLC cable (Figure 3C). The angular orientation of these ppMLC cables was predominantly orthogonal to the prospective folding axis of the epithelium tissue, which ran circumferentially about 200 µm from the micropattern edges (Figure 3D). This observed angular orientation of apical ppMLC was similar to that observed by Nishimura et al., whereby pMLC was enriched mediolaterally to form linear cables that were oriented perpendicularly to the anterior–posterior axis along which the neural plate folds.^[^ ^33^, ^34^ ^]^ We then examined the role of PCP pathway which is important in mediating apical constriction during neurulation.^[^ ^33^ ^]^ Dishevelled‐2 (DVL2) is an intracellular Wnt‐signaling mediator, which is known to play a key role in PCP when they are preferentially localized to actin fibers instead of the cell cytoplasm.^[^ ^35^ ^]^ DVL2 expression at the apical domain of the folding epithelium of 56‐h old ME‐primed μNET was colocalized with ppMLC (Figure 3F). However, since the angular distribution of DVL2 on the apical tissue surface could not be determined due to imaging access limitation, it remains unclear if PCP pathway was directly driving tissue folding in ME‐primed μNETs. Nonetheless, we observed that prospective NE cells near the hinge region of the 3D fold in the ME‐primed μNETs were significantly elongated (Figure 3F(ii),G) and exhibited the characteristic wedge‐shape cell morphology of a bending NE (Figure 3F(iii),H). There was also preferential localization of phospho‐histone 3 (PH3)^+^ mitotic cells at the apical side of the bending region of the prospective NE tissue (Figure 3I,J).^[^ ^36^, ^37^ ^]^ This resembled the pseudo‐stratified columnar‐like epithelial cells in a developing neural plate.^[^ ^33^ ^]^ The above results suggest that spatially‐juxtaposed ME cells could promote NE tissue organization through physiologically‐relevant morphogenetic processes.

### 2.4 Mesoendoderm Supports Primitive Ectoderm Bending Through TGF*β*‐Mediated Signaling

**Figure 4:**
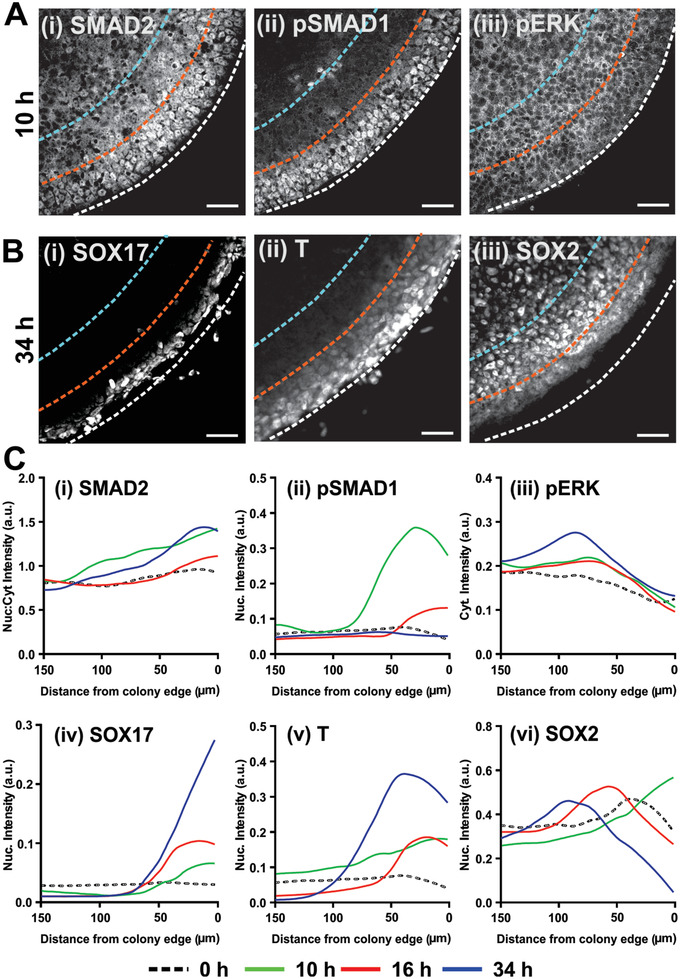
Spatio‐temporal patterning of intracellular signaling and cell lineages preceded 3D tissue morphogenesis in ME‐primed μNET. A,B) Immunofluorescence images showing localization patterns of Smad1, Smad2, and ERK signaling and lineage markers in H9 derived *μ*P‐hPSC colony periphery. A) *μ*P‐hPSC colonies after 10 h of ME induction exhibited (i) nuclear localization of SMAD2 near the colony periphery and cytoplasmic localization toward the colony interior, (ii) a strong band of nuclear pSMAD1 at the colony periphery, and (iii) relatively homogeneous expression of pERK along the edge and interior of the colony. B) *μ*P‐hPSC colonies at 34 h after differentiation exhibited spatial patterning of germ lineage markers (i) SOX17, (ii) T, (iii) SOX2. Dotted lines in (A) and (B) denote 50 µm (orange) and 100 µm (blue) radial distance from colony edges (white). C) Averaged spatio‐temporal profiles of (i) SMAD2 nuclear:cytoplasmic (Nuc:cyt) intensity ratio, (ii) nuclear pSMAD intensity, (iii) cytoplasmic pERK intensity, (iv) nuclear SOX17 intensity, (v) nuclear T intensity, and (vi) nuclear SOX2 intensity as a function of time after induction of *μ*P‐hPSCs colonies. Data are average ± s.e.m of three colonies from two independent experiments. Scale bars in (A) and (B) = 50 µm.

Next, we seek to understand how ME cells coordinated tissue bending in the adjacent primitive ectoderm. Spontaneously differentiated micropatterned hPSCs have been shown to induce germ layer patterning via spatial localization of bone morphogenetic protein (BMP), transforming growth factor beta (TGF*β*), and fibroblast growth factor (FGF) signaling.^[^ ^10^ ^]^ Therefore, we monitored the spatio‐temporal dynamics of these signaling pathways and cell fate specification in the micropatterned hPSC colony when it was still a 2D monolayer (0–34 h) (**Figure** **4**, Figure S4, Supporting Information). Spatial patterning of SMAD1, SMAD2, and ERK was established immediately after the first 10 h of ME priming (Figure 4A,C(i,ii)). Upon withdrawal of exogenous BMP4, SMAD1 activity decreased rapidly to baseline levels by 34 h (Figure 4C(ii)); whereas SMAD2 and pERK activities persisted (Figure 4C(i,iii)). The emergence of cell fate patterning in the micropatterned colonies was most apparent at 34 h post differentiation, where spatial distributions of T^+^, SOX17^+^, and SOX2^+^ cells correlated to SMAD1, SMAD2, and ERK activations respectively (Figure 4B,C). Direct NE induction in the unprimed μNETs did not result in any Smad1 and Smad2 activation, which correlated with the absence of T^+^ and SOX17^+^ cell fates after 34 h of differentiation (Figure S4, Supporting Information). Our data indicated that spatial patterning of ME and prospective NE cells emerged while the micropatterned hPSC colony was still a 2D monolayer, and their interactions may drive subsequent morphogenetic processes to give rise to the laminar folded structures, which was confirmed using live‐cell imaging (Video S2, Supporting Information).

**Figure 5:**
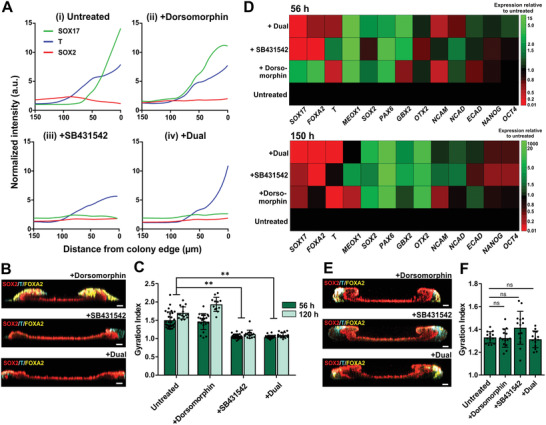
Specification of endodermal cells by TGF signaling supported prospective NE tissue folding. A) Spatial expression profiles of SOX17, T, and SOX2 in micropatterned H9 derived hPSC colonies at 34 h after differentiation in (i) untreated controls or in the presence of (ii) 2 µm Dorsormorphin; (iii) 1 µm SB431542; (iv) 2 µm Dorsormorphin + 1 µm SB431542. All inhibitors were added after 10 h of ME induction. B,C) Inhibition of TGF*β* signaling initiated at 10 h post induction resulted in the specific abrogation of endodermal (FOXA2^+^) lineages, which was accompanied by attenuation of tissue folding. B) Cross‐sectional view of 3D confocal sections of whole 56‐h old ME‐primed μNETs treated with inhibitors. C) Gyration indices of 56‐h and 120‐h old ME‐primed μNETs. Data are average ± s.e.m of at least eight colonies from three independent experiments. D) Heat map displaying transcriptional profiles of 56‐h old and 150‐h old ME‐primed μNETs treated with different inhibitors initiated at 10 h after induction relative to untreated control samples. Transcript levels are average of three independent experiments. E,F) Addition of inhibitors at 34 h after induction when patterning of three germ lineages have been established did not affect tissue folding. E) Cross‐sectional views of 3D confocal sections of whole 56‐h old ME‐primed μNETs treated with inhibitors, and corresponding F) gyration indices indicative of tissue folding. Data in (F) are average ± s.e.m of 7 colonies from two independent experiments. Asterisks indicate statistical significance (One‐way ANOVA followed by Tukey´s post‐test, **p < 0.0001; n.s. indicates no statistical significance). Scale bars in (B) and (E) = 100 µm.

To ascertain the roles of ME cells in modulating the morphogenic folding of the primitive ectoderm, we added pharmacological inhibitors of BMP (Dorsomorphin) and TGF*β* signaling (SB431542) immediately after the 10‐h ME induction phase. Addition of Dorsomorphin did not attenuate the initial specification of T^+^ cells at the colony edge (**Figure** **5**A(ii)) as expected from the low level of endogenous pSMAD1 activity at 34 h (Figure 4C(i)). In contrast, SB431542 treatment alone or in combination with Dorsomorphin resulted in the specific abrogation of SOX17^+^ cells from the colony periphery at 34 h (Figure 5A(iii,iv), indicating that endogenous TGF*β* signaling was required for specification of SOX17^+^ endodermal cells. We followed the morphogenetic processes and found that TGF*β* inhibition more potently attenuated the folding of the μNET structures than BMP inhibition (Figure 5B). The gyration index, which is commonly employed to measure the extent of neural tissue folding in vivo,^[^ ^38^ ^]^ of SB431542‐treated μNETs were significantly lower than that of untreated colonies; whereas those treated with Dorsomorphin were not significantly affected (Figure 5C, Figure S5A, Supporting Information). Regardless of the resultant tissue architecture, prolonged BMP and TGF*β* inhibitions led to an enrichment of neural‐specific transcripts^[^ ^27^ ^]^ while attenuating mesoderm and endoderm‐specific transcripts respectively (Figure 5D). When Dorsomorphin or SB431542 treatment was initiated only at 34 h after spatial patterning of SOX2^+^, T^+^, and SOX17^+^ cells in the hPSC micropatterns was already established, we observed that the morphogenic folding process was not significantly affected (Figure 5E,F). The same phenomenon was observed with both H9 and H1 hPSC lines (Figure S5B, Supporting Information). The results indicated that TGF*β* signaling must be sustained for a sufficient duration to support the development of endodermal cell population, which in turn modulated the folding of the adjacent pre‐NE tissue.

### 2.5 Modeling Genetically‐Induced Defects in NE Morphogenesis

**Figure 6:**
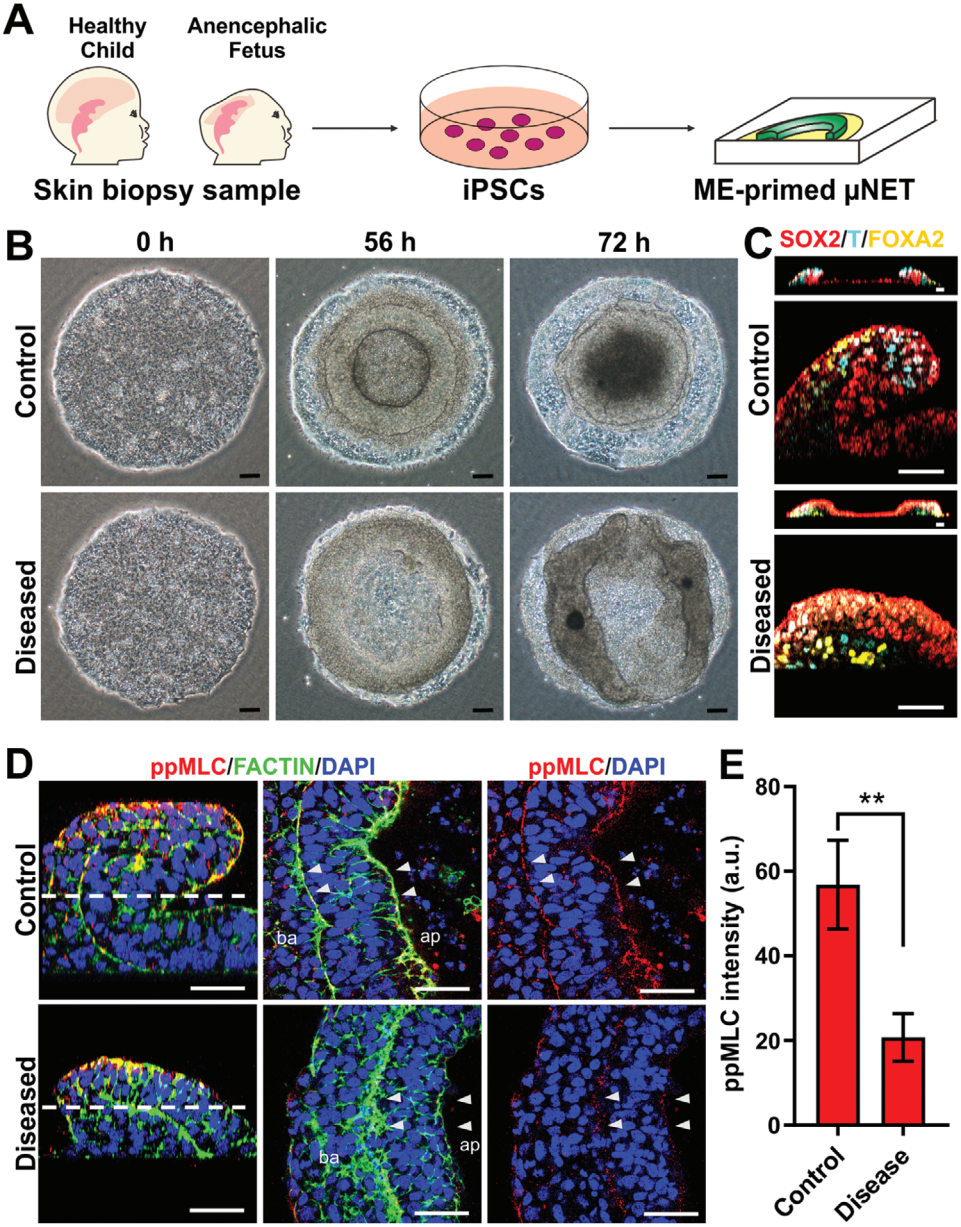
NTD iPSC displayed abrogated prospective NE tissue folding in ME‐primed μNETS which was mediated by defective actomyosin contractile machinery. A) Schematics depicting formation of ME‐primed μNETs from iPSCs generated by reprogramming of primary dermal fibroblasts from anencephalic fetus (diseased) and a healthy ethnically matched child (control) into. B) Gross structural morphologies of ME‐primed μNETs generated using control iPSCs and diseased iPSCs. C) Localization patterns of germ layer markers: endoderm (FOXA2), mesoendoderm (T), and primitive ectoderm/neuroepithelium (SOX2) in 56‐h old ME‐primed μNETs generated from control iPSCs and diseased iPSCs. Images are cross‐sectional views of 3D confocal sections (top panel) and magnified cross‐sectional side views of μNETs (bottom panel) illustrate a loss of 3D folding in the diseased μNETs as compared to control μNETs while retaining trilaminar germ layer organization. D) Expression patterns of apical constriction markers, ppMLC, and F‐actin in ME‐primed 56 h‐old ME‐primed μNETs generated from control iPSCs and diseased iPSCs. Images of magnified cross‐sectional side views and single optical section transversing the μNET structure displaying disorganization of F‐actin and loss of ppMLC cable in disease derived μNETs as compared to control μNETs. E) Quantification of ppMLC intensity in control and disease μNETs. Data are average of ± s.e.m of six slices from three independent samples (Student's *t*‐test, **p < 0.0001). Scale bars in (B) = 100 µm and (C,D) = 50 µm.

We then assessed whether the emergent NE tissue structure in ME‐primed μNETs can be used as a readout to detect neurodevelopmental abnormalities. Specifically, we selected genetic mutations associated with two human neurodevelopmental syndromes, which have known molecular targets relevant to NE morphogenesis. Anencephaly is a form of NTD, which can be caused by a deleterious recessive mutation in NUAK2, serine/threonine kinase that results in impaired actomyosin contractility via the Hippo‐YAP pathway.^[^ ^39^ ^]^ induced pluripotent cells (iPSCs) were derived from anencephalic non‐viable fetuses born to consanguineous parents bearing the NUAK2 deletion by Sendai virus reprogramming as previously reported^[^ ^40^ ^]^ (**Figure** **6**A and Figure S6, Supporting Information). A control iPSC line was also generated from a healthy ethnically‐matched child (Figure 6A and Figure S6, Supporting Information). When ME‐primed μNETs were generated from the iPSC lines, we could observe an obvious structural anomaly in the anencephalic μNET as early as 56 h post differentiation as compared to healthy control (Figure 6B). Although the laminar organization of the three germ layers were present in both the anencephalic and healthy μNETs, the diseased μNET did not exhibit any tissue folding (Figure 6C). Since anencephalic iPSC have defective actomyosin machineries,^[^ ^39^ ^]^ we proceeded to examine whether the absence of tissue folding in the diseased μNET was attributed to apical constriction dysfunction. The basal to apical views revealed that F‐actin bundles were expanded and disorganized at the interface between ME and the prospective NE, and were not properly assembled at the apical surface in diseased μNETs (Figure 6D). This was accompanied by significant reduction in expression of ppMLC in diseased μNETs compared to control micropatterns (Figure 6D,E). The disruption in actomyosin contractile cables at the apical surface of the prospective NE tissue and its interface with the adjacent ME tissue suggested that autosomal genetic mutation in the anencephalic patient caused an anomaly in apical constriction mediated pathways to result in a structural defect, which can be easily detected in the ME‐primed μNET model.

**Figure 7:**
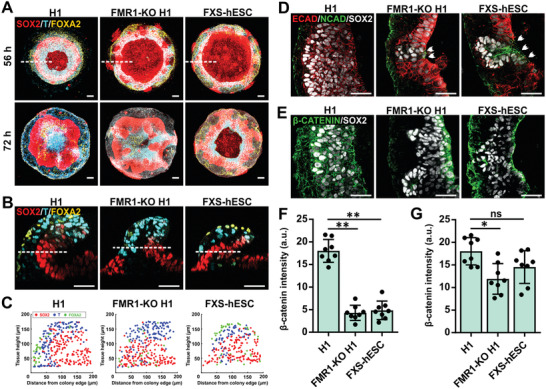
FMR1 silencing in Fragile X Syndrome resulted in μNET structural dysmorphia that was mediated by cadherin and *β*‐catenin dysfunctions. A–C) Localization patterns of germ layer markers: endoderm (FOXA2), mesoendoderm (T), and neuroepithelium (SOX2) in ME‐primed μNETs generated from normal H1 human embryonic stem cell, an isogenic FMR1 knock‐out H1 cell line (FMR1‐KO H1) and a FXS human embryonic stem cell line (FXS‐hESC). A) Maximum intensity projections of 3D confocal sections of 56‐h old (top panel) and 72‐h old (bottom panel) μNETs generated from normal and diseased cell lines. B) Magnified cross‐sectional views of 3D confocal sections. C) Localization map of SOX2^+^, T^+^ and FOXA2^+^ cells along *x*‐*z* cross‐sectional plane indicated by white dotted lines in (A), illustrate a loss of laminar organization of SOX2^+^, T^+^, and FOXA2^+^ cells in the FMR1‐KO‐H1 and FXS‐hESC μNETs as compared to normal H1 μNETs. Data are overlay map of >3 optical sections from two independent experiments. D,E) Magnified views of single optical sections transversing 56‐h old ME‐primed μNETs generated using control and diseased cell lines along the dotted white lines in (B) showing the expression patterns of FMRP targets, namely N‐cadherin (NCAD), E‐cadherin (ECAD), and *β*‐catenin relative to SOX2. D) In FMR1‐KO‐H1 and FXS‐hESC μNETs, regions with SOX2^+^ NE cell migration was accompanied by premature ECAD downregulation and NCAD upregulation indicated by white arrows. E) *β*‐catenin was preferentially lost from the SOX2^+^ NE layers in the FMR1‐KO‐H1 and FXS‐hESC μNETs; whereas *β*‐catenin was uniformly expressed in the normal H1 μNETs. F,G) Quantification of *β*‐catenin expression levels in ME‐primed μNETs generated using normal and diseased cell lines in E) SOX2^+^ cells, F) SOX2^−^ cells. Data are average of ± s.e.m of eight optical sections from three independent experiments. Asterisks indicate statistical significance (one‐way ANOVA followed by Tukey´s post‐test, *p < 0.005, **p < 0.0001). Scale bars in (A) = 100 µm, (B–D) = 50 µm.

Fragile X Syndrome (FXS) is one of the most common autism spectrum neurodevelopmental disorders caused by epigenetic silencing of the Fragile X mental retardation 1 (*FMR1*) gene.^[^ ^41^ ^]^ This results in the downregulation of the RNA‐binding protein fragile X mental retardation protein (FMRP), which in turn regulates the translation of many target proteins that are upstream mediators of *β*‐catenin, including NCAD^[^ ^42^ ^]^ and ECAD.^[^ ^43^ ^]^ Since both ECAD and NCAD are present and spatially organized in the μNET model, we postulate that pathological effects of *FMR1* mutation in FXS can be manifested as morphological aberrations. To this end, we generated ME‐primed μNETs using normal H1 human embryonic stem cell, an isogenic *FMR1* knock‐out H1 cell line (FMR1‐KO H1) and a FXS human embryonic stem cell line (FXS‐hESC).^[^ ^44^ ^]^The FMR1‐KO‐H1 and FXS‐hESC lines were verified for complete absence of FMRP expression using immunoblotting. (Figure S7A, Supporting Information). In ME‐primed μNETs, we could not identify consistent transcriptional changes in the diseased μNETs that can be attributed to *FMR1* mutation (Figure S7B, Supporting Information), which was similar to previous studies reporting that normal and FXS‐hESC undergoing directed neuronal differentiation could not be distinguished at the NE stage.^[^ ^45^ ^]^ However, analysis of the μNET structures by immunocytochemistry revealed that the trilaminar germ layer organization was lost in the *FMR1*‐KO H1 and FXS μNETs due to regionalized invasion of SOX2^+^ NE cells into the surrounding ME tissues (**Figure** **7**A–C, Figure S7C, Supporting Information).

Subsequently, the μNETs were characterized for the spatial localization of *β*‐catenin and its upstream regulators (i.e., NCAD, ECAD). We found that in regions where we observed NE invasion into the lateral ME tissues, there was premature ECAD‐to‐NCAD switching in the SOX2^+^ cells (Figure 7D). We also discovered that *β*‐catenin was preferentially downregulated in the SOX2^+^ cells of *FMR1*‐KO H1 and FXS μNETs (Figure 7E,F) as compared to *β*‐catenin levels in the SOX2^−^ cells (Figure 7E,G). The results are suggestive that downregulation of FMRP‐deficiency in FMR1‐KO H1 and FXS μNETs resulted in the loss of *β*‐catenin specifically in the prospective NE, which destabilized the tissue architecture as evident by sporadic regional premature E‐to‐NCAD switching, accompanied by the acquisition of a migratory cellular phenotype. This demonstrates that deficits in neurogenesis due to loss of FMRP in FXS can be detected right at the onset of neurodevelopment through aberrations in NE tissue morphogenesis.

### 2.6 Modeling Drug‐Induced Defects in NE Morphogenesis

**Figure 8:**
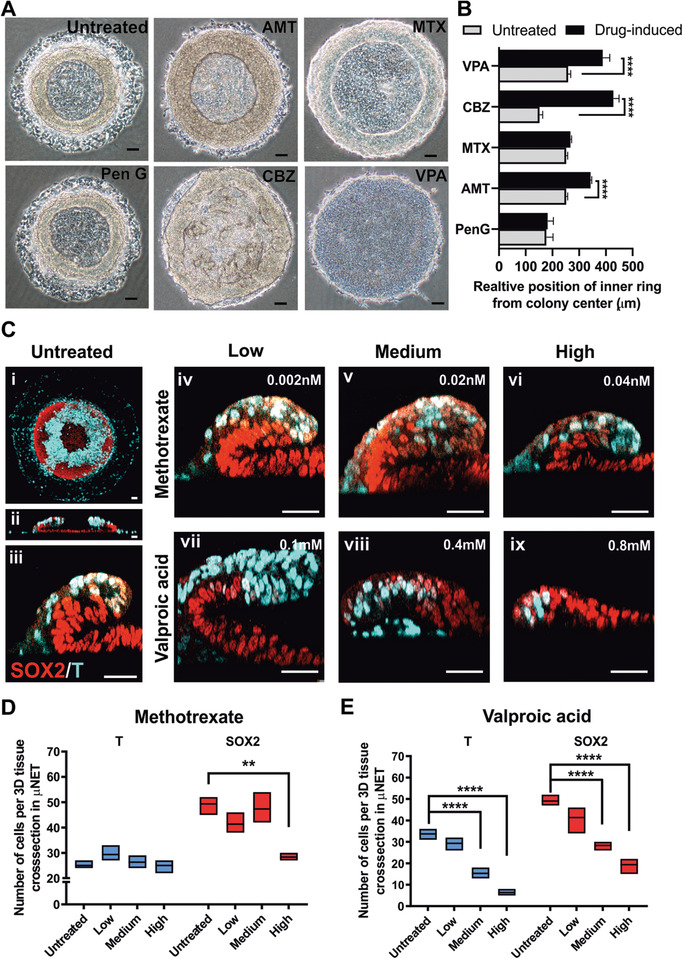
Disruption of emergent tissue structures in ME‐primed μNETs by NTD‐inducing drugs. A) Gross structural morphologies of ME‐primed μNETs generated in absence or presence of highest test concentration of four NTD‐inducing drugs, including two specific FAA (MTX and AMT) and two non‐specific FAAs (VPA and CBZ) and a non‐NTD inducing control drug, penicillin G. B) Quantification of relative position of inner ring of annular structure from the colony center, used to measure morphological aberration, in ME‐primed μNETs on treatment with control and NTD‐inducing drugs. C) Immunofluorescence labeling of pre‐NE marker, SOX2, and mesoendoderm marker, T in 56‐h old untreated and NTD‐inducing drug treated μNETs (i) Maximum projection of a 3D confocal section of the entire untreated μNET. Cross‐sectional views of 3D confocal sections of (ii) whole μNETs and (iii–ix) magnified regions where 3D tissue folding occurred in absence and presence of NTD‐inducing drug. D,E) Quantification of T^+^ and SOX2^+^ cells in the magnified regions where 3D tissue folding occurred in absence and presence of NTD‐inducing drug. B) Data are average of ± s.e.m of ≥ 15 independent measurements from *n* = 2, D,E) Data are average of ± s.e.m of four cross sections from two independent samples. ****p < 0.00005, ***p < 0.0005, **p < 0.005, *p < 0.05 (ANOVA). Scale bars (A) = 100 µm, (C) = 50 µm. Methotrexate (MTX), Aminopterin (AMT), Valproic acid (VPA), Carbamazepine (CBZ), Penicillin G (Pen G). Following IC_25_ values, cell lines used for respective drugs are H1 for MTX, AMT, CBZ and PenG, and H9 for VPA.

Finally, we assessed whether the ME‐primed μNET could be used as a screening platform for NTD inducing drugs and discern their associated mechanisms of neuropathogenesis. Many drugs are known to cause or increase the risk of NTDs in human either by specifically antagonizing a component of the folic acid metabolic pathway (thereafter referred to as specific folic acid antagonists, FAA) or non‐specifically affecting folic acid metabolism as well as other embryonic developmental pathways (referred to as non‐specific FAA).^[^ ^46^, ^47^ ^]^ A panel of four NTD‐inducing drugs, consisting of two specific FAA (methotrexate and aminopterin) and two non‐specific FAAs (valproic acid and carbamazepine) as well as a non‐NTD inducing control drug, penicillin G, were evaluated for their dose‐dependent effects on the emergent tissue structure of ME‐primed μNETs. The drug concentrations were selected to be below the IC_25_ values of the respective drugs on H1 and/or H9 cell lines (Figure S8 and Table S2, Supporting Information) in order to observe disruption of cell differentiation and morphogenetic processes at non‐cytotoxic conditions. Since the μNET displayed a distinct emergent tissue structure in the form of a 3D annular ring at 56 h, we first evaluated for gross morphological changes of the annular ring when the μNETs were generated in the presence of the drugs. It was observed that Penicillin, the non‐NTD inducing control drug, did not alter the resultant tissue structure in the μNET model (**Figure** **8**A,B). In contrast, the non‐specific FAAs (carbamezapine and valproic acid) could elicit apparent disruption to the gross 3D annular structures; whereas the effect of specific FAAs (methotrexate and aminopterin) were more subtle (Figure 8A,B and Figure S9, Supporting Information). However, when we mapped out the organization of ME and prospective NE cell populations in the folding primitive ectoderm of drug‐treated μNETs, there were obvious changes in the emergent tissue organization in both specific and non‐specific FAA treatment groups (Figure 8C). In the presence of a non‐specific FAA drug, such as valproic acid, the 3D invagination‐like tissue folding was completely disrupted at medium and high drug concentrations as a result of a significant reduction in both T^+^ ME and SOX2^+^ pre‐NE cell populations (Figure 8C(iv–vi),D). Specific FAAs, exemplified by methotrexate, did not completely abrogate the 3D folding even at high concentrations, which would explain the lack of observable gross morphological change to the 3D annular ring (Figure 8C(vii–ix)). However, it was observed that the drug preferentially attenuated the proliferation of SOX2^+^ pre‐NE cells over T^+^ ME cells (Figure 8E). Interestingly, these results indicated that methotrexate likely preferentially targeted NE development over other lineages due to the critical role of folate metabolism in neural tube development.^[^ ^48^ ^]^ In contrast, valproic acid is an embryotoxic drug,^[^ ^49^ ^]^ which does not select between ME and NE development. This highlights the capability of μNET model can differentiate between different classes of NTD inducing drugs.

## 3 Discussion

We showed that patterning ME cells within a geometrically confined hPSC colony can be used to direct the self‐organization of a developing NE tissue, which recapitulates neurodevelopment‐relevant morphogenetic cellular processes. A key advantage of our approach is that it offers an exceptional degree of control over the formation of the emergent NE tissue architecture as compared to spontaneously self‐organized neural rosettes in conventional EBs or monolayer cultures.^[^ ^1^ ^]^ We can direct the geometry and the folding dynamics of the μNETs by changing the size and shape of the initial hPSC micropattern as well as the duration of ME induction. By precisely tuning these parameters, we can generate NE tissues with defined architectures with an unprecedented level of consistency down to <1.5% variation between different colonies. This has important implications in the practical translation of the μNETs. In the context of disease modeling and drug testing, the realization of uniform tissue structures will provide a stable baseline against which effects of environmental (e.g., teratogens) or genetic factors can be evaluated. In addition, the μNETs are easily scalable as tissue arrays in multi‐well plate format, and are also amenable to high content imaging. A caveat is that the tissues must remain adherent to the culture substrates, which lasts ≈6 days since progressive folding of the NE results in a highly contractile tissue that detaches from the culture substrate.

We exploited the consistency at which we can direct NE tissue folding to probe the underlying cellular mechanisms in which ME orchestrates tissue folding. We unveil that TGF*β* signaling mediated by SOX17^+^ endoderm cells played a more crucial role in inducing folding of the primitive ectoderm (the prospective NE) than BMP signaling (Figure 5C–E). Multiple studies in various developmental models have implicated BMP antagonism in neural plate folding.^[^ ^50^, ^51^, ^52^ ^]^ It is only until recently that the role of TGF*β* as the upstream regulator of BMP activity at the medial hinge point is being elucidated in chick embryos.^[^ ^53^ ^]^ A similar role of TGF*β* signaling in mediating NE folding is mirrored in our ME‐primed μNET model, thereby providing unique opportunities to uncover how the TGF‐BMP signaling cascades intersect with the apicobasal polarity pathways within the context of human neurodevelopment.

Finally, we demonstrated that evaluating structural dysmorphia uniquely achievable with the μNET model allows us to model pathological processes associated with human neurodevelopmental defects. μNETs generated from iPSCs derived from a human anencephalic patient robustly manifested a defect in NE tissue folding that was mediated by dysfunctions in the apical constriction machinery (Figure 6). Therefore, μNETs generated from either patient‐derived or genetically edited hPSCs provide a human‐specific experimental model to functionally validate candidate NTD genes identified from either human transcriptomics and epigenomics profiling studies or transgenic mice models.^[^ ^54^ ^]^ In addition, we also demonstrated that by examining gross morphology and cellular organization of the emergent folding tissue structure in the μNET model, we could successfully identify paradigm NTD‐inducing drugs from a negative control drug (penicillin) (Figure 8). More importantly, this model could differentiate between NTD‐inducing drugs, which specifically affected NE development by interfering with folate metabolism (e.g., methotrexate), from non‐specific FAA drugs, which targeted both ME and NE development (e.g., valproic acid). Due to the scalability, consistency, and relatively short duration in which we can generate the μNETs, it will be an ideal to screen for environmental factors, such as folate, glucose, and drugs, which can cause NTDs directly or indirectly through interactions with genetic factors.

In the context of neurodevelopmental diseases, such as FXS, with later neurodevelopmental etiologies involving neural network maturation and plasticity,^[^ ^41^ ^]^ the μNET model can serve as an early screening model for dysfunctions of known molecular targets that have pleiotropic functions in different stages of neural development. This is exemplified with the early detection of abnormalities in ECAD and NCAD through structural aberrations in μNETs derived from FXS‐hESC and *FMR1*‐KO H1 lines (Figure 7). Although the μNET is only representative of early neurodevelopment and may not be able recapitulate all FMRP targets in the context of neural network maturation, which is more relatable to clinical symptoms of FXS, it is likely that the effect of *FMR1* silencing in FXS can be manifested in different cellular processes during the course of development. Due to the scalability, consistency and relatively short duration in which we can generate the μNETs, it will be an ideal screening platform to rank order potential therapeutic pharmacological agents or gene editing constructs before lead candidates are selected for further validation.^[^ ^55^ ^]^ Since NTDs and many monogenic neurodevelopmental diseases, for example, FXS, Rett syndrome manifest structural deformities in the embryonic nervous system,^[^ ^56^ ^]^ we expect that the μNETs will be equally applicable in facilitating breakthroughs in identifying therapies to correct for such neurodevelopmental disorders.

## Experimental Section

##### Experimental Model and Subject Details—Cell Lines

The human embryonic stem cell line used for the experiments were H9 and H1 that were obtained from WiCell Research institute, Inc. (Madison, WI, USA) and confirmed to be contamination free. Primary fibroblasts from anencephalic fetus and a healthy ethnically matched child (control) were reprogrammed into iPSC were generated using retroviral vectors or using the CytoTune‐iPS 2.0 Sendai Reprogramming Kit (Thermo Fisher Scientific, A16517), as previously reported in,^[^ ^40^ ^]^ in accordance with the manufacturer's instructions. For FXS diseased cell lines, patient derived FXS hESC (WCMC‐37) was obtained from Dr. Nikica Zaninovic in Weill Cornell Medical College, New York and FMR1 KO hESC (knockout FXS cell line) was generated by CRISPR/Cas9 targeting exon 3 of *FMR1* gene, in the H1 hESC background^[^ ^44^ ^]^

##### Experimental Model and Subject Details—Maintenance of Human Pluripotent Stem Cells

The basement membrane matrix used to culture and maintain the cells was hPSC‐qualified Matrigel (354 277, BD Biosciences) and the maintenance medium was mTeSR1 medium (05850, StemCell Technologies). To passage the cells, the 80% confluent hPSC culture was enzymatically treated with Dispase (07923, StemCell Technologies) followed by mechanical scrapping to select only the undifferentiated colonies for every passage.

##### Method Details—Fabrication of Stencils for Micropatterning

The stencils for cell micropatterning were made using Polydimethylsiloxane (PDMS). The components of PDMS stencil comprised of a thin PDMS sheet ≈130 µm and thick 2 mm PDMS gasket (Specialty Silicone Products Inc.) and were designed using AutoCAD. The thin PDMS sheet was synthesized by spin coating the liquid uncured PDMS (SYLGARD 184, Dow Corning Co., USA) on a salinized glass slide and then baked at 60 °C for 3–4 h. A 2 mm thick PDMS sheet (Specialty Silicone Products Inc., USA) was laser‐cut to a rectangular gasket, which was then bonded with the thin PDMS sheet using thin layer of liquid uncured PDMS (SYLGARD 184, Dow Corning Co., USA) followed by baking at 60 °C for 3–4 h to seal the assembly of thick gasket and thin PDMS sheet. The complete assembly was carefully removed from the salinized glass slide and punched to generate circular through‐holes of appropriate size to finally get the PDMS stencil for cell micropatterning. For generating PDMS stencils with different shapes, the thin PDMS sheets were laser cut using CO_2_ laser to generate through‐holes of desired shape. Before being used for hPSC micropatterning, the stencil was sterilized by autoclaving at 120 °C for 30 min.

##### Method Details—Generation of Micropatterned hPSC Colonies

The autoclaved PDMS stencil was sealed onto a 60 mm tissue culture polystyrene dish (Nunc) with 200 µL 70% analytical grade ethanol in milliQ water and then the dish was placed inside biosafety cabinet for ethanol to dry. After the stencil was temporarily sealed on the petri dish, the stencil was treated with air plasma for 60 s to decrease the hydrophobicity of PDMS stencil. Then, the stencil was coated with 1.5X hPSC‐qualified Matrigel solution and incubated for 5 h at 37 °C. For micropatterning hPCSs, a single cell suspension of hPSCs was obtained by enzymatic treatment of confluent and undifferentiated H9 cells with Accutase (SCR005, Merck Millipore) for about 8 min. The single hPSC cells were suspended in mTeSR1 medium supplemented with 10 µm Y27632 (72 304, Calbiochem, Merck Millipore) and were later seeded onto the Matrigel‐coated PDMS stencils at a density of 4444 cells mm^−2^ to make a confluent layer of cells on the PDMS stencil. The cells were incubated for 1 h to allow proper attachment with the Matrigel‐coated substrate. After successful attachment of cells, the PDMS stencil was removed leaving behind the micropatterns of hPSC that could sieve through the through holes of the stencil and attached to the substrate. The unpatterned areas of the substrate were passivated with 0.5% Pluronic F‐127 (P2443, Sigma‐Aldrich) in DMEM/F12 for 10 min. The hPSC micropatterns were later washed thrice with DMEM/F12 and supplemented with mTeSR1 medium containing 10 µm Y27632 for at least 5 h. The differentiation of hPSC micropatterns was then initiated after 24 h of cell seeding for subsequent time.

##### Method Details—Induction of Micropatterned hPSC Colonies

To induce mesoendodermal differentiation, cells were cultured in basal STEMdiff APEL2 medium (05270, StemCell Technologies) supplemented with 100 ng mL^−1^ Activin A (338‐AC‐025, R&D Systems), 25 ng mL^−1^ BMP4 (314‐BP‐010, R&D Systems), and 10 ng mL^−1^ FGF2 (233‐FB‐025, R&D Systems). To induce neuroectoderm differentiation, the cells were cultured in neural induction medium N2B27 that was formulated by mixing DMEM/F12+GlutaMAX (10 505 018, GIBCO), neurobasal medium (1 749 130, GIBCO), 0.5X B27 supplement (A1486701, GIBCO), 0.5X N2 supplement (07152, GIBCO), 0.1 mm *β*‐mercaptoethanol (21 985 023, Gibco) and 0.2 mm l‐Glutamine (25 030 081, GIBCO). For experiments involving SMADs inhibitions, 2 µm Dorsomorphin (P5499, Sigma‐Aldrich) and/or 1 µm SB431542 (72 234, StemCell Technologies) were supplemented in the neural induction medium after 10 or 34 h of differentiation according to experimental time points described in the main text.

##### Method Details—Immunofluorescence Staining and Imaging

The samples were fixed for 20 min in 3.7% paraformaldehyde at room temperature, and permeabilized for 24 h in 1% Triton X‐100 solution in sterile PBS at 4 °C on an orbital shaker followed by 24 h incubation in blocking buffer (2% BSA and 1% Triton X‐100 in sterile PBS) at 4 °C on an orbital shaker. After overnight incubation, the samples were incubated with primary antibodies diluted in antibody dilution buffer (2% BSA, 0.2% Triton‐X 100 in sterile PBS) at 4 °C for 48 h. The primary antibodies used in this study were SOX2 (mouse, MAB2018, R&D Systems, 1:50), SOX2 (goat, AF2018, R&D Systems, 1:30), Brachyury (goat, AF2085, R&D Systems, 1:50), Brachyury (rabbit, MAB20851, R&D Systems, 1:50), ECAD (rabbit, sc‐7870, Santa Cruz, 1:50), NCAD (mouse, ab19348, Abcam, 1:100), Nestin (rabbit, ab92391, Abcam, 1:200), pSMAD1 (rabbit, 9516S, cell signaling technology, 1:100), SMAD2 (mouse, 610 842, BD biosciences, 1:150), pERK (rabbit, 4695P, Cell signaling technology, 1:500), ppMLC (rabbit, 3674S, cell signaling technology, 1:100), ZO1 (mouse, 33‐9100, Thermofisher scientific, 1:100), SOX17 (goat, AF1924, R&D Systems, 1:50), FOXA2 (mouse, sc‐374375, Santa Cruz, 1:50), GBX2 (mouse, SAB1403854, Sigma, 1:100), NCAM (rabbit, AB5032, Merck, 1:100), *β*‐catenin (mouse, sc‐7963, Santa Cruz, 1:50), NANOG (goat, AF1997, R&D Systems, 1:200), OCT4 (mouse, 111 351, Santa Cruz, 1:200), SSEA4 (mouse, MAB4304, Millipore, 1:200), TRA‐1‐81 (mouse, MAB4381, Millipore, 1:200), TRA‐1‐60 (mouse, MAB4360, Millipore, 1:200). The samples were then washed with washing buffer (3% NaCl and 0.2% Triton‐X 100 in sterile PBS) for 1 h at room temperature for two times followed by incubation in washing buffer on an orbital shaker at 4 °C for 24 h. Then the samples were incubated with secondary antibodies, 0.5 µg mL^−1^ DAPI (Invitrogen) and Alexa Fluor 488 phalloidin (A12379, Thermofisher scientific, 1:200) (if required) at 4 °C for 24 h on an orbital shaker. The secondary antibodies used in this study were donkey anti‐rabbit IgG‐Alexa Fluor 488 (1:500, Life Technologies), donkey anti‐rabbit IgG‐Alexa Fluor 647 (1:500, Life Technologies), donkey anti‐mouse IgG‐Alexa Fluor 555 (1:500, Life Technologies), donkey anti‐goat IgG‐Alexa Fluor 546 (1:500, Life Technologies), donkey anti‐goat IgG‐Alexa Fluor 633 (1:500, Life Technologies). The samples were then washed following similar washing steps as after primary antibody incubation. After that, samples were mounted using RapiClear 1.47 (Sunjin lab) pre‐warmed to 37°C to facilitate tissue clearing. The samples were prepared by placing 0.2 or 0.5 mm deep spacer around the sample followed by sealing with a coverslip. The immunofluorescence images were acquired using LSM800 confocal laser scanning system (Zeiss). Time lapse imaging was performed using ESID detection system capturing a total of 330 frames with every frame after 4 min using LSM800 confocal laser scanning system (Zeiss) equipped with temperature and CO_2_ control.

##### Method Details—Immunobloting

Cells were lysed with RIPA buffer (Sigma Aldrich) containing complete Protease Inhibitor cocktail tablets (Roche). Protein concentration was measured using the Bradford assay (BioRad). The samples were denatured at 70 °C for 10 min in 4× NuPAGE sample buffer and 10× NuPAGE reducing agent (Thermo Fisher). A total of 30 µg of protein per sample was separated on 4–12% bis‐tris gradient gels in MOPS SDS running buffer (Thermo Fisher) at 100 V for 3 h followed by transfer to nitrocellulose membrane at 120 V for 1.5 h at room temperature. The following primary antibodies were used for detection: anti‐FMRP (MAB2160, Millipore) and anti‐Calnexin (Sigma, C4731). Alexa‐Fluor 680 goat anti‐mouse (Thermo Fisher) and DyLight 800 goat anti‐rabbit (Rockland) were used as secondary antibodies. Membranes were imaged using the Li‐Cor Odyssey infrared imaging system

##### Method Details—Quantitative Polymerase Chain Reaction

RNA was isolated from μNETs using RNeasy Mini kit and RNeasy Micro kit (Qiagen) accordingly. RNA concentration of each sample was determined using NanoDrop (Thermo Scientific). Reverse transcription to cDNA was performed using Tetro cDNA Synthesis Kit (Bioline). Quantitative PCR was performed using FastStart Universal SYBR Green Master (ROX) (Roche) on Stratagene MX3000P (Agilent Technologies). Primers sequences used in PCR are listed in Table S1, Supporting Information.

##### Method Details—Transcriptional Profiling

For transcriptional profiling, the heatmaps were generated with MATLAB built in function. The colormap of each heatmap was transformed using a custom MATLAB code. Briefly, for each set of gene expression data, the values were sorted and their empirical cumulative distribution function was computed. The colormap was then indexed on the increments of the cumulative distribution function instead of the gene expression values. Visually, it procures an equal color separation between all the data points.

##### Method Details—Preparation of Drugs and Drug Treatment

The drugs used for evaluating drug induced neuropathogenesis were Valproic Acid (P4543‐10G, Sigma‐Aldrich), Methotrexate (13 960, Cayman Chemical), Aminopterin (ALX‐440‐041‐M050, Enzo Lifesciences), Carbamazepine (298 464, Carbosynth) and Penicillin G (13752‐1G‐F, Sigma‐Aldrich). The stocks of Methotrexate, Aminoprotein and Carbamazepine were prepared by dissolving in dimethyl sulfoxide (D2650, Sigma‐Aldrich), while Valproic acid and Penicillin G was dissolved in sterile Milli‐Q water. The stocks prepared for Methotrexate, Aminoptrein, Carbamazepine and Valproic acid were 100 nm, 50 nm, 8464 µm, and 625 mm, respectively. For test the effect of drugs on μNET model, three test concentrations (designated as low, medium and high) were selected based on cytotoxicity levels of each drug on H1 cell lines and the respective concentrations were added to the differentiation medium at each step of the protocol, including the daily change of differentiation medium.

The cytotoxicity test was performed using MTS assay in 96‐well plates format. Each drug was tested with 8 serial concentrations with five times dilution factor along with solvent and negative controls. Each well was plated with 10 000 hPSCs and were subjected to 72 h drug treatment with daily change of medium supplemented with drugs. After 72 h, the cell viability was measured using CellTiter 96 AQueous One Solution Cell Proliferation Assay (MTS, G3581, Promega). IC25 values were then calculated using regression model or manually using GraphPad Prism 7.

##### Quantification and Statistical Analysis—Image Quantification

A custom MATLAB code was designed to automatically analyze the acquired fluorescence intensity values from multi‐channeled image datasets. All sample images sets to be quantified were fed automatically into the code and stacks of the individual channels were stored. The DAPI channel was first processed to define nuclei center positions using a set series of image processing methods which includes contrast equalization (adapthisteq) filtering (weiner2), thresholding (graythresh), and finally, binary operations (bwmorph and imopen). Overlapping nuclei were differentiated using a watershed approach (watershed). Centroids of each nuclei were then obtained using MATLAB command region props. For each of the centroids, circles of fixed radius (5 pixels or 3.12 µm) were digitally constructed and used as a binary mask to extract and quantify intensity values from the individual channel image slices of the corresponding sample. Non‐zero pixel values were then averaged (labeled Nuc) to obtain the mean intensity value for a particular nucleus. To obtain mean intensity values from the cytosolic region of each cell (labelled Cyt) instead, the masks that were constructed were subsequently morphologically dilated (imdilate) by a factor of two using a disk morphological structuring element (strel) and the original nuclei mask was subtracted from this new larger mask. Also, from centroidal information, the distance of each cell from the periphery of the colony was calculated, allowing the visualization of intensity across each tissue sample as a 1D plot. To average intensity values across samples, locally weighted scatterplot smoothening (https://blogs.mathworks.com/loren/2011/01/13/data-driven-fitting/) with fixed interspaced points was used to cater for the difference in spatial positioning of the centroids. For 2D trilaminar organization visualization, each cell was classified as SOX2+, T+ or SOX17+ based on mean intensity value ratio comparison.

##### Quantification and Statistical Analysis—Statistical Analysis

The experiments were performed as biological triplicates, unless mentioned otherwise, and the results were generated as mean with standard error. For data sets with only two comparison groups, Student's *t*‐test was performed to determine the statistical significance and for multiple comparison groups, one‐way ANOVA analysis followed by Tukey‐Kramer Multiple Comparisons Test.

## Conflict of Interest

The authors declare no conflict of interest.

## Author Contributions

G.S. and Y.C.T. designed research; G.S. performed majority experiments, C.S.Y. performed RT‐PCR experiments, intensity profile quantifications, and some research experiments, G.S., C.S.Y., and Y.C.T. analyzed data, G.S. and J.T.C.M. performed data quantification, J.Z.Y.T. helped in drug treatment experiments, J.J.C.F. designed MATLAB code for heatmaps, C.B., P.W.C., T.T.T., U.A., and H.K. provided diseased cell lines; B.R. and M.P. supported diseased cell line experiments, and G.S. and Y.C.T. wrote the paper.

## Supporting information

Supporting Information

Supplemental Video 1

Supplemental Video 2

## References

[1] M. Simunovic , A. H. Brivanlou , Development 2017, 144, 976. 28292844 10.1242/dev.143529 PMC5358114

[2] J. A. Palmer , A. M. Smith , L. A. Egnash , K. R. Conard , P. R. West , R. E. Burrier , E. L. R. Donley , F. R. Kirchner , Birth Defects Res., Part B 2013, 98, 343. 10.1002/bdrb.21078 24123775

[3] S. Kameoka , J. Babiarz , K. Kolaja , E. Chiao , Toxicol. Sci. 2014, 137, 76. 24154490 10.1093/toxsci/kft239

[4] J. M. Panzica‐Kelly , K. C. Brannen , Y. Ma , C. X. Zhang , O. P. Flint , L. D. Lehman‐Mckeeman , K. A. Augustine‐Rauch , Toxicol. Sci. 2013, 131, 447. 23042729 10.1093/toxsci/kfs293

[5] F. Uibel , M. Schwarz , Reprod. Toxicol. 2015, 55, 30. 25263227 10.1016/j.reprotox.2014.09.009

[6] P. R. West , A. M. Weir , A. M. Smith , E. L. R. Donley , G. G. Cezar , Toxicol. Appl. Pharmacol. 2010, 247, 18. 20493898 10.1016/j.taap.2010.05.007

[7] J. A. Boos , P. M. Misun , A. Michlmayr , A. Hierlemann , O. Frey , Adv. Sci. 2019, 6, 1900294. 10.1002/advs.201900294 PMC6662399 31380185

[8] Y.‐C. Toh , J. Xing , H. Yu , Biomaterials 2015, 50, 87. 25736499 10.1016/j.biomaterials.2015.01.019

[9] C. L. Bauwens , R. Peerani , S. Niebruegge , K. A. Woodhouse , E. Kumacheva , M. Husain , P. W. Zandstra , Stem Cells 2008, 26, 2300. 18583540 10.1634/stemcells.2008-0183

[10] A. Warmflash , B. Sorre , F. Etoc , E. D. Siggia , A. H. Brivanlou , Nat. Methods 2014, 11, 847. 24973948 10.1038/nmeth.3016 PMC4341966

[11] X. Xue , Y. Sun , A. M. Resto‐Irizarry , Y. Yuan , K. M. Aw Yong , Y. Zheng , S. Weng , Y. Shao , Y. Chai , L. Studer , J. Fu , Nat. Mater. 2018, 17, 633. 29784997 10.1038/s41563-018-0082-9 PMC6622450

[12] T. Haremaki , J. J. Metzger , T. Rito , M. Z. Ozair , F. Etoc , A. H. Brivanlou , Nat. Biotechnol. 2019, 37, 1198. 31501559 10.1038/s41587-019-0237-5

[13] M. J. Harris , D. M. Juriloff , Birth Defects Res., Part A 2005, 73, 532. 10.1002/bdra.20170 15968625

[14] M. R. Brouns , H. W. van Straaten , Drug Discovery Today: Dis. Models 2005, 2, 285.

[15] S. Nasr Esfahani , Y. Shao , A. M. Resto Irizarry , Z. Li , X. Xue , D. L. Gumucio , J. Fu , Biomaterials 2019, 216, 119244. 31207406 10.1016/j.biomaterials.2019.119244 PMC6658735

[16] J. Xing , Y. Cao , Y. Yu , H. Li , Z. Song , H. Yu , Sci. Rep. 2017, 7, 8491. 28819231 10.1038/s41598-017-09178-1 PMC5561212

[17] J. Xing , Y.‐C. Toh , S. Xu , H. Yu , Sci. Rep. 2015, 5, 10038. 25966467 10.1038/srep10038 PMC4428054

[18] Z. Ma , J. Wang , P. Loskill , N. Huebsch , S. Koo , F. L. Svedlund , N. C. Marks , E. W. Hua , C. P. Grigoropoulos , B. R. Conklin , K. E. Healy , Nat. Commun. 2015, 6, 7413. 26172574 10.1038/ncomms8413 PMC4503387

[19] M. L. Iruela‐Arispe , G. J. Beitel , Development 2013, 140, 2851. 23821032 10.1242/dev.070680 PMC3699276

[20] E. Nikolopoulou , G. L. Galea , A. Rolo , N. D. E. Greene , A. J. Copp , Development 2017, 144, 552. 28196803 10.1242/dev.145904 PMC5325323

[21] Y. Zheng , X. Xue , A. M. Resto‐Irizarry , Z. Li , Y. Shao , Y. Zheng , G. Zhao , J. Fu , Sci. Adv. 2019, 5, eaax5933. 31844664 10.1126/sciadv.aax5933 PMC6905871

[22] A. Ranga , M. Girgin , A. Meinhardt , D. Eberle , M. Caiazzo , E. M. Tanaka , M. P. Lutolf , Proc. Natl. Acad. Sci. U. S. A. 2016, 113, E6831. 27742791 10.1073/pnas.1603529113 PMC5098636

[23] Z. Koledova , in 3D Cell Culture: Methods and Protocols (Ed: Z. Koledova ), Springer, New York, NY 2017, pp. 107–124.

[24] A. E. Hegab , D. Arai , J. Gao , A. Kuroda , H. Yasuda , M. Ishii , K. Naoki , K. Soejima , T. Betsuyaku , Stem Cell Res. 2015, 15, 109. 26042794 10.1016/j.scr.2015.05.005

[25] I. Martyn , T. Y. Kanno , A. Ruzo , E. D. Siggia , A. H. Brivanlou , Nature 2018, 558, 132. 29795348 10.1038/s41586-018-0150-y PMC6077985

[26] Q.‐L. Ying , M. Stavridis , D. Griffiths , M. Li , A. Smith , Nat. Biotechnol. 2003, 21, 183. 12524553 10.1038/nbt780

[27] S. M. Chambers , C. A. Fasano , E. P. Papapetrou , M. Tomishima , M. Sadelain , L. Studer , Nat. Biotechnol. 2009, 27, 275. 19252484 10.1038/nbt.1529 PMC2756723

[28] Y. Tao , S.‐C. Zhang , Cell Stem Cell 2016, 19, 573. 27814479 10.1016/j.stem.2016.10.015 PMC5127287

[29] J. M. Halbleib , W. J. Nelson , Genes Dev. 2006, 20, 3199. 17158740 10.1101/gad.1486806

[30] A. Dady , C. Blavet , J.‐L. Duband , Dev. Dyn. 2012, 241, 1333. 22684994 10.1002/dvdy.23813

[31] F. Fagotto , Development 2014, 141, 3303. 25139853 10.1242/dev.090332

[32] J. M. Sawyer , J. R. Harrell , G. Shemer , J. Sullivan‐Brown , M. Roh‐Johnson , B. Goldstein , Dev. Biol. 2010, 341, 5. 19751720 10.1016/j.ydbio.2009.09.009 PMC2875788

[33] T. Nishimura , H. Honda , M. Takeichi , Cell 2012, 149, 1084. 22632972 10.1016/j.cell.2012.04.021

[34] E. M. Mcgreevy , D. Vijayraghavan , L. A. Davidson , J. D. Hildebrand , Biol. Open 2015, 4, 186. 25596276 10.1242/bio.20149589 PMC4365487

[35] C. P. Nielsen , K. K. Jernigan , N. L. Diggins , D. J. Webb , J. A. Macgurn , Cell Rep. 2019, 28, 1074. 31340145 10.1016/j.celrep.2019.06.083 PMC6884140

[36] Y. Zhu , M. Carido , A. Meinhardt , T. Kurth , M. O. Karl , M. Ader , E. M. Tanaka , PLoS One 2013, 8, e54552. 23358448 10.1371/journal.pone.0054552 PMC3554725

[37] L. Subramanian , M. Bershteyn , M. F. Paredes , A. R. Kriegstein , Nat. Commun. 2017, 8, 14167. 28139695 10.1038/ncomms14167 PMC5290330

[38] S. Budday , C. Raybaud , E. Kuhl , Sci. Rep. 2014, 4, 5644. 25008163 10.1038/srep05644 PMC4090617

[39] C. Bonnard , N. Navaratnam , K. Ghosh , P. W. Chan , T. T. Tan , O. Pomp , A. Y. J. Ng , S. Tohari , R. Changede , D. Carling , B. Venkatesh , U. Altunoglu , H. Kayserili , B. Reversade , J. Exp. Med. 2020, 217, e20191561. 32845958 10.1084/jem.20191561 PMC7953732

[40] P. H. Chia , F. L. Zhong , S. Niwa , C. Bonnard , K. H. Utami , R. Zeng , H. Lee , A. Eskin , S. F. Nelson , W. H. Xie , S. Al‐Tawalbeh , M. El‐Khateeb , M. Shboul , M. A. Pouladi , M. Al‐Raqad , B. Reversade , eLife 2018, 7, e32451. 29784083 10.7554/eLife.32451 PMC5963920

[41] T. Halevy , C. Czech , N. Benvenisty , Stem Cell Rep. 2015, 4, 37. 10.1016/j.stemcr.2014.10.015 PMC4297868 25483109

[42] G. La Fata , A. Gärtner , N. Domínguez‐Iturza , T. Dresselaers , J. Dawitz , R. B. Poorthuis , M. Averna , U. Himmelreich , R. M. Meredith , T. Achsel , C. G. Dotti , C. Bagni , Nat. Neurosci. 2014, 17, 1693. 25402856 10.1038/nn.3870

[43] R. Lucá , M. Averna , F. Zalfa , M. Vecchi , F. Bianchi , G. L. Fata , F. Del Nonno , R. Nardacci , M. Bianchi , P. Nuciforo , S. Munck , P. Parrella , R. Moura , E. Signori , R. Alston , A. Kuchnio , M. G. Farace , V. M. Fazio , M. Piacentini , B. De Strooper , T. Achsel , G. Neri , P. Neven , D. G. Evans , P. Carmeliet , M. Mazzone , C. Bagni , EMBO Mol. Med. 2013, 5, 1523. 24092663 10.1002/emmm.201302847 PMC3799577

[44] K. H. Utami , N. H. Skotte , A. R. Colaço , N. A. B. M. Yusof , B. Sim , X. Y. Yeo , H.‐G. Bae , M. Garcia‐Miralles , C. I. Radulescu , Q. Chen , G. Chaldaiopoulou , H. Liany , S. Nama , U.‐K. A. Peteri , P. Sampath , M. L. Castrén , S. Jung , M. Mann , M. A. Pouladi , Biol. Psychiatry 2020, 88, 500. 32653109 10.1016/j.biopsych.2020.05.005

[45] M. Telias , M. Segal , D. Ben‐Yosef , Dev. Biol. 2013, 374, 32. 23219959 10.1016/j.ydbio.2012.11.031

[46] S. Hernández‐Díaz , M. M. Werler , A. M. Walker , A. A. Mitchell , N. Engl. J. Med. 2000, 343, 1608. 11096168 10.1056/NEJM200011303432204

[47] S. Hernandez‐Diaz , Am. J. Epidemiol. 2001, 153, 961. 11384952 10.1093/aje/153.10.961

[48] A. E. Beaudin , P. J. Stover , Birth Defects Res., Part A 2009, 85, 274. 10.1002/bdra.20553 PMC4435943 19180567

[49] C. Wegner , H. Nau , Neurology 1992, 42, 17. 1574172

[50] P. Ybot‐Gonzalez , C. Gaston‐Massuet , G. Girdler , J. Klingensmith , R. Arkell , N. D. E. Greene , A. J. Copp , Development 2007, 134, 3203. 17693602 10.1242/dev.008177

[51] D. S. Eom , S. Amarnath , J. L. Fogel , S. Agarwala , Development 2011, 138, 3179. 21750029 10.1242/dev.058602 PMC3133910

[52] C. M. Mizutani , N. Meyer , H. Roelink , E. Bier , PLoS Biol. 2006, 4, e313. 16968133 10.1371/journal.pbio.0040313 PMC1563485

[53] S. Amarnath , S. Agarwala , J. Cell Sci. 2017, 130, 119. 27034139 10.1242/jcs.179192 PMC5394770

[54] J. B. Wallingford , L. A. Niswander , G. M. Shaw , R. H. Finnell , Science 2013, 339, 1222002. 23449594 10.1126/science.1222002 PMC3677196

[55] E. M. Shitik , A. A. Velmiskina , A. A. Dolskiy , D. V. Yudkin , Gene Ther. 2020, 27, 247. 32203197 10.1038/s41434-020-0141-0

[56] H. Sontheimer , in Diseases of the Nervous System, Academic Press, San Diego 2015, pp. 319–347.

