# A Micropatterned Human‐Specific Neuroepithelial Tissue for Modeling Gene and Drug‐Induced Neurodevelopmental Defects

**DOI:** 10.1002/advs.202101786

**Published:** 2021-07-07

**Authors:** Geetika Sahni, Shu‐Yung Chang, Jeremy Choon Meng Teo, Jerome Zu Yao Tan, Jean Jacques Clement Fatien, Carine Bonnard, Kagistia Hana Utami, Puck Wee Chan, Thong Teck Tan, Umut Altunoglu, Hülya Kayserili, Mahmoud Pouladi, Bruno Reversade, Yi‐Chin Toh

*Adv. Sci*. **2021**, *8*, 2001100

DOI: 10.1002/advs.202001100

In the originally published article, the first name and family name of the third author were mixed‐up. The corrected author byline is presented here.

