# Supplementary material for: A Micropatterned Human‐Specific Neuroepithelial Tissue for Modeling Gene and Drug‐Induced Neurodevelopmental Defects
Source: Adv Sci (Weinh). 2021 Jan 6;8(5):2001100. doi: 10.1002/advs.202001100 (PMC7927627; doi:10.1002/advs.202001100)
Supplement: Supplementary file 1 — Supporting Information [file ADVS-8-2001100-s001.pdf]

© 2020 Wiley-VCH GmbH

ADVANCED SCIENCE
Open Access

Supporting Information

for *Adv. Sci.,* DOI: 10.1002/advs.202001100

A micropatterned human-specific neuroepithelial tissue for modeling gene and drug-induced neurodevelopmental defects

*Geetika Sahni, Shu-Yung Chang, Jeremy Teo Choon Meng, Jerome Zu Yao Tan, Jean Jacques Clement Fatien, Carine Bonnard, Kagistia Hana Utami, Puck Wee Chan, Thong Teck Tan, Umut Altunoglu, Hülya Kayserili, Mahmoud Pouladi, Bruno Reversade, and Yi-Chin Toh**

Supplementary information

Sahni et al., A micropatterned human-specific neuroepithelial tissue for modeling gene and drug-induced neurodevelopmental defects

Supplementary Figures

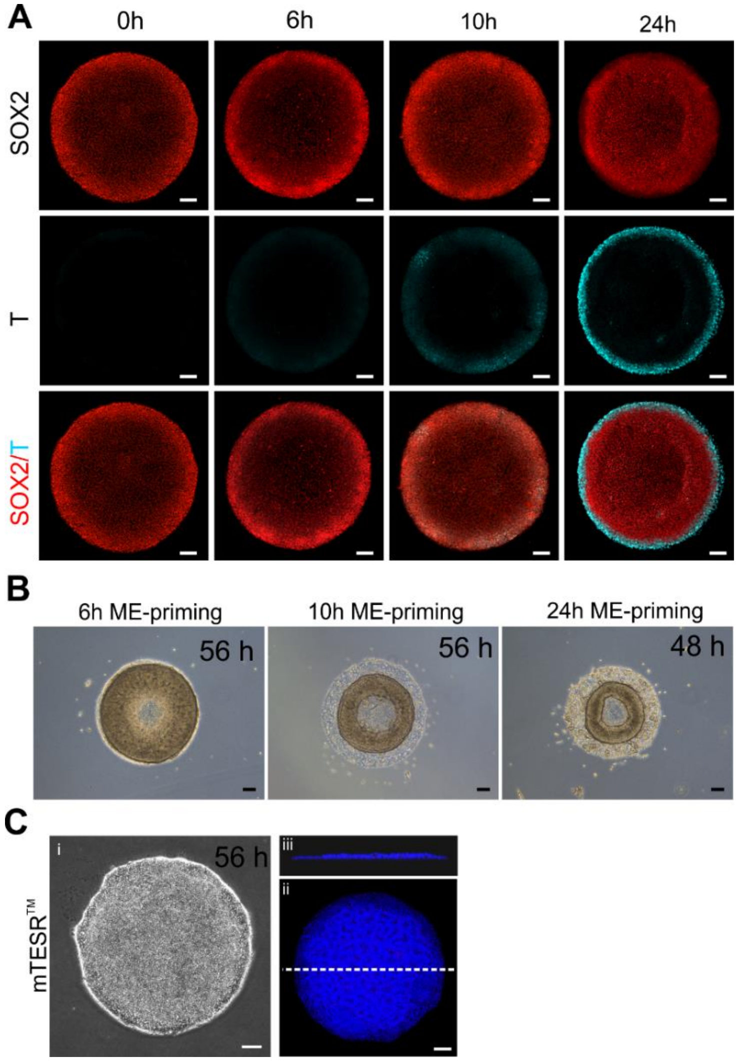

SI Figure. 1. *Duration of mesoendoderm (ME) priming controls the spatial patterning and dynamics of H9 derived ME-primed μNET formation. (A) Immunofluorescence staining of ME marker, Brachyury (T) as well as the pluripotency/ neuroepithelium marker, SOX2, in micropatterned-hPSCs colonies after 0, 6, 10, and 24 hours of ME induction. T expression at the colony periphery could only be detected only after 10 hours of ME induction. (B) Phase contrast images of ME-primed μNET structures formed with 6, 10 and 24 hours of ME induction followed by neuroepithelium induction until the earliest time point at which 3D annular structure was formed. (C) Phase and fluorescent DAPI images of hPSC micropatterns grown in stem cell renewal mTESR^{TM} medium for 56 hours do not show and folding pattern as observed in μNETS. Scale bars = 100 μm.*

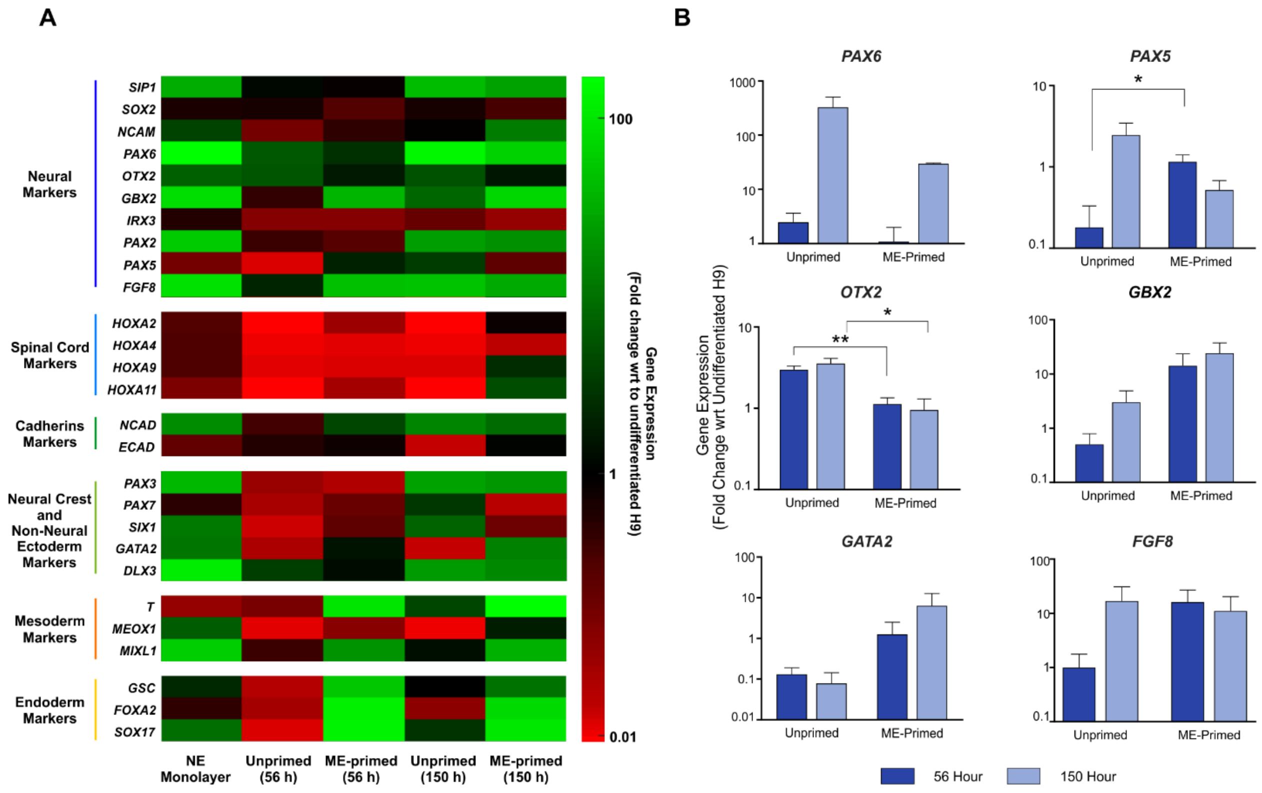

SI Figure. 2. *(A) Transcriptional profiling of H9 derived unprimed and ME-primed μNETs at 56 and 150 hours after differentiation along with reference expressions in unpatterned NE monolayer at 150 hours. Transcript levels are normalized to undifferentiated hPSCs, H9. (B) Independent transcriptional profiles of neural markers, PAX6, PAX5, OTX2, GBX2, FGF8 and non-neural ectoderm marker GATA2. Data are average ± s.e.m of at least 2 independent experiments. (Student's t-test, *p<0.05, **p<0.01).*

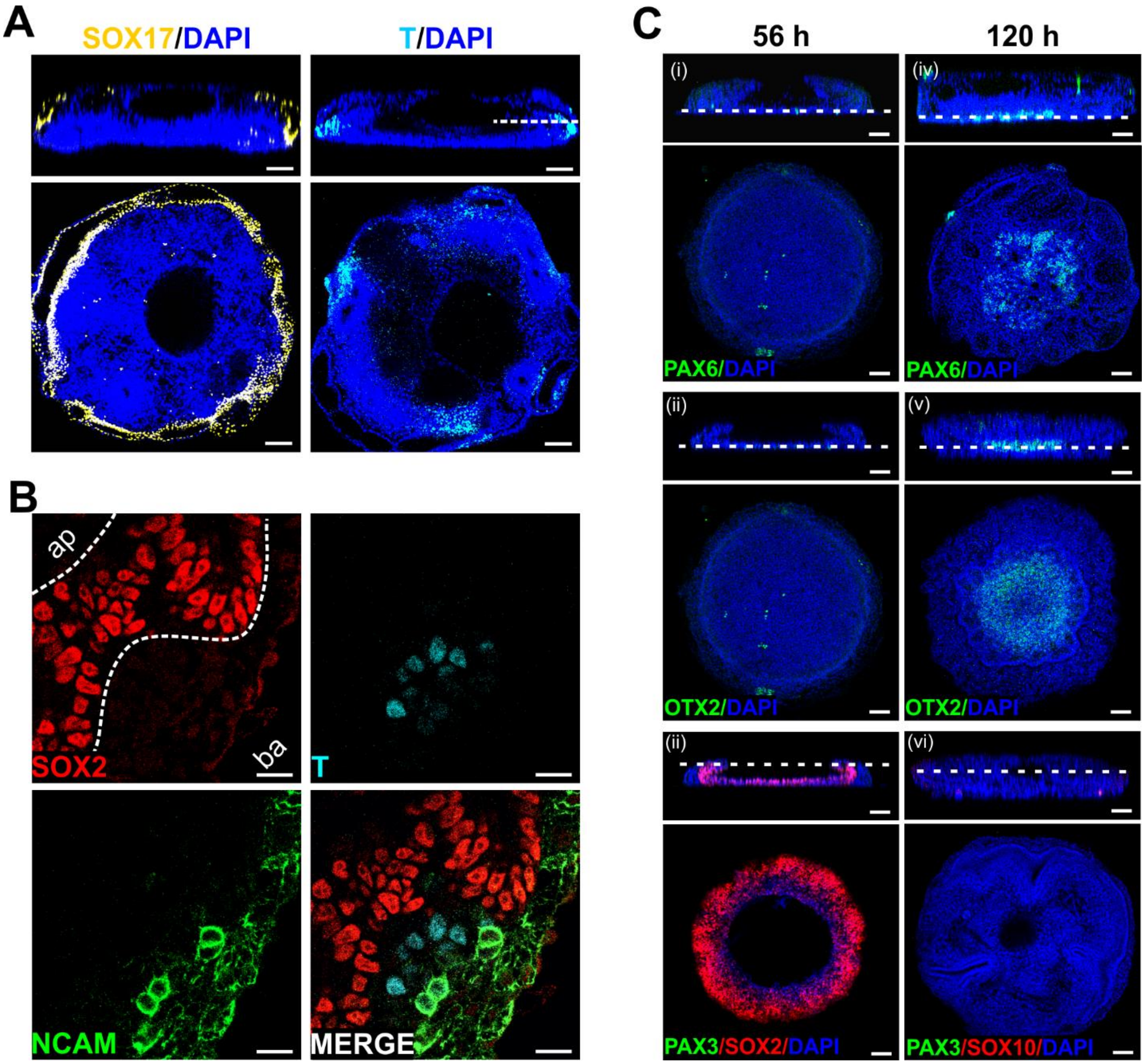

SI Figure. 3. *Immunofluorescence labeling of neural and non-neural markers in H9-derived ME-primed μNETs. (A) Maximum intensity projections (bottom panels) and cross-sectional views (top panels) of 3D confocal sections of whole ME-primed μNETs showed that endoderm SOX17$^+$ cells were localized to the boundaries of the 3D NE structure whereas mesoendoderm T$^+$ cells were localized to the tip of the 3D fold in 120-hour old ME-primed μNETs. (B) Magnified view of single optical sections transversing the ME-primed μNETs along the dotted white lines in (A). It was observed that NCAM$^+$ cells marking for neuroepithelium were segregated from T$^+$ mesoendodermal cells at 120 hours post differentiation while still localizing basal to the SOX2$^+$ cells. (C) Expression patterns of neuroepithelium markers (PAX6 and OTX2) and neural crest and non-neural ectoderm markers (PAX3 and SOX10) in (i-iii) 56-hour old ME-primed μNET and (iv-vi) 120-hour old ME-primed μNETs. Images are cross-sectional views of*

3D confocal sections of whole ME-primed μNETs (top panels) and of single optical sections transversing the μNETs along the dotted white lines (bottom panel). ap-apical, ba-basal. Scale bars in (A-C) = 100 μm, (B) = 20 μm

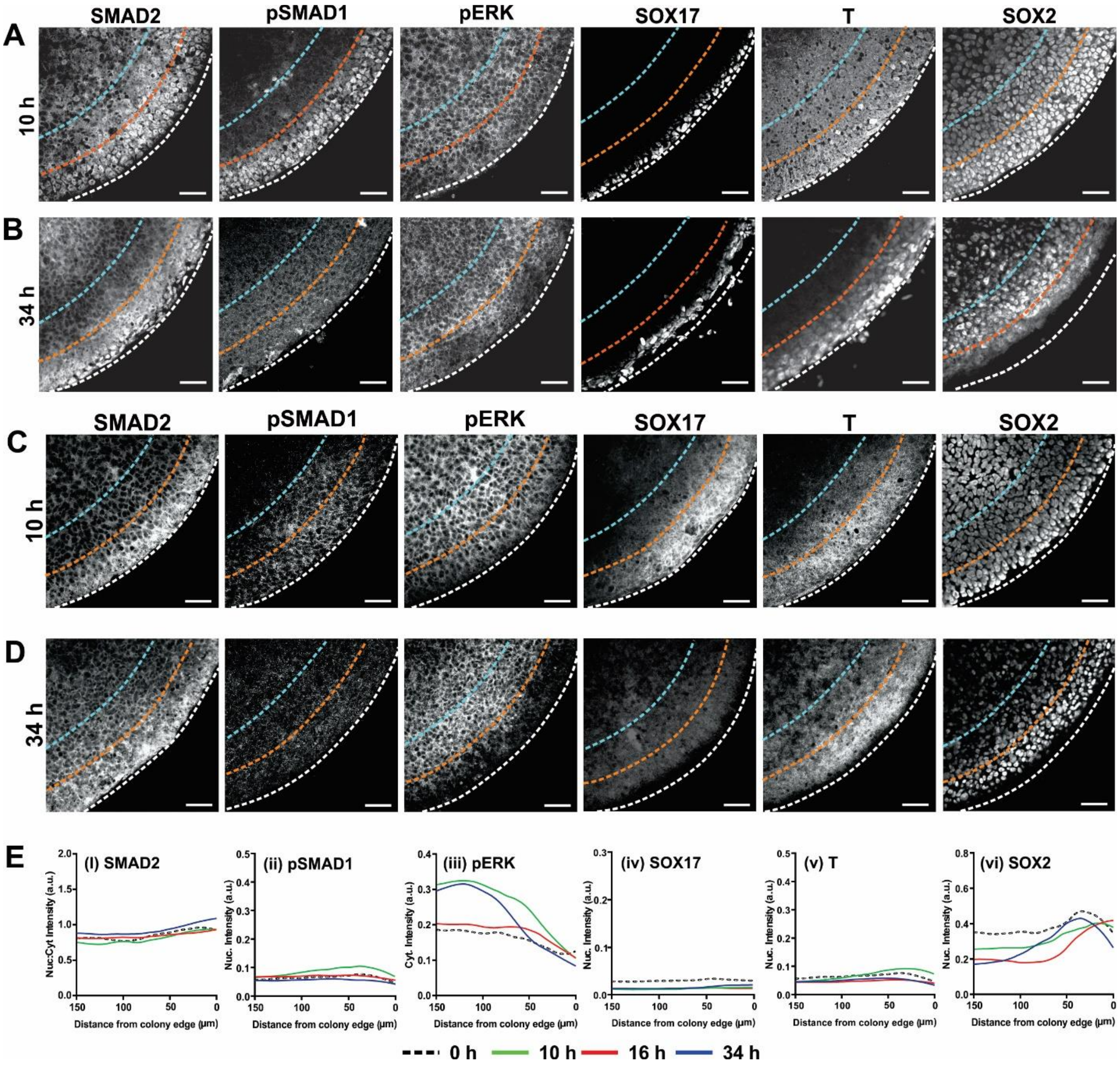

SI Figure. 4. *Spatio-temporal patterning of intracellular signaling and lineage markers in H9 derived (A-B) ME-primed and (C-E) unprimed μNET at different time-points after differentiation. (A-D) Immunofluorescence images of colony edge of μP-hPSCs staining for signaling molecules, SMAD2, pSMAD1, pERK and germ lineage markers, SOX17, T, SOX2 in μP-hPSC colonies. (A) After 10 hours of mesoendoderm induction, preferential activation of SMAD2 and SMAD1 signaling corresponded to nuclear expression of SOX17 as a narrow band along the colony periphery. There was weak expression of T at the colony periphery, and pERK along with SOX2 expression were relatively uniform at the edge and interior of the colony. (B)*

At 34 hours after differentiation, there was nuclear expression of SMAD2 but not pSMAD1 along the colony periphery. pERK was present at ~ 100 μm from the colony edge. (C) after 10 hours of direct neuroepithelium induction, SMAD2, cytoplasmic pERK and nuclear SOX2 were homogeneously expressed along the edge and interior of the colony, whereas no expression was detected for pSMAD1, SOX17 and T throughout the colony. (D) The expression patterns at 34 hours were similar to that after 10 hours of direct neuroepithelium induction, except an increase in the expression of pERK at ~ 100 μm from the colony edge. Dotted lines in (A-D) denote 50 μm (orange) and 100 μm (blue) radial distance from colony edges (white). (E) Averaged spatio-temporal expression profiles of signaling molecules and lineage markers in unprimed μNET as a function of time after induction of μP-hPSCs colonies. (i) SMAD2 nuclear:cytoplasmic (Nuc:cyt) ratio; (ii) nuclear pSMAD intensity; (iii) cytoplasmic pERK intensity; (iv) nuclear SOX17 intensity; (v) nuclear T intensity; and (iv) nuclear SOX2 intensity. Data are average of ± s.e.m of 3 colonies from 2 individual experiments. Scale bars in (A-D) = 50 μm.

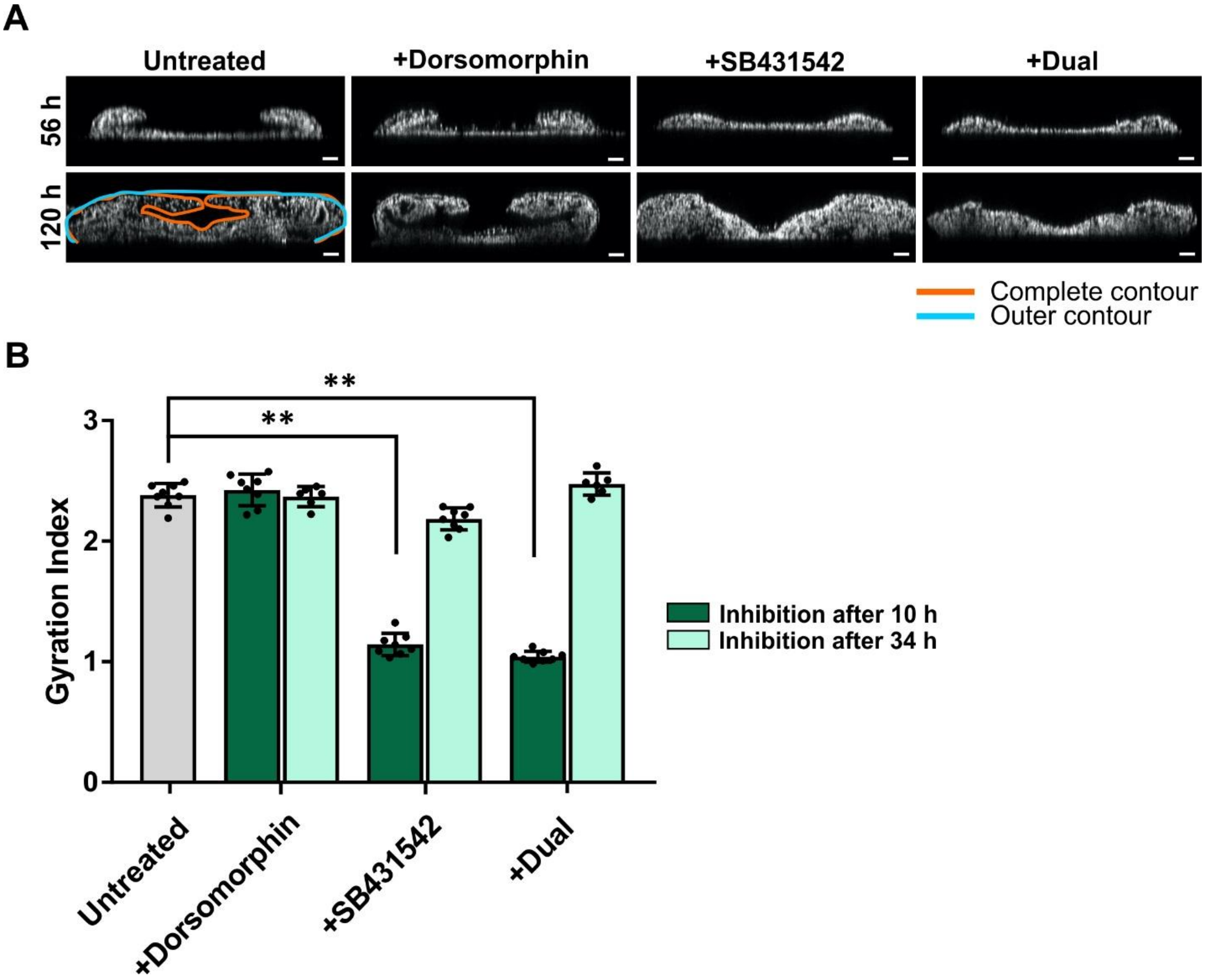

SI Figure. 5. *Extent of neural tissue folding was determined by measuring the gyration index. (A) H9 derived ME-primed µNETs at 56 and 120 hours post differentiation in the presence of BMP and TGFβ inhibitors. 2 µM Dorsormorphin, 1 µM SB431542 or both inhibitors were added at 10 hours after ME induction. Tissue samples were stained using DAPI to label the cell nuclei and orthogonal projections of 3D confocal sections were acquired. The gyration index is defined as the ratio of the complete contour (orange lines) / outer contour (blue line) (B) Effect of BMP and TGFβ inhibitors on 3D morphogenesis in ME-primed µNETs differentiated from H1 human embryonic stem cells (WiCell). Quantification of 3D folding, as measured by gyration index, in 120-hour old ME-primed µNETs differentiated from H1 human embryonic stem cells (WiCell) formed in the absence (Untreated) or presence of 2 µM Dorsormorphin (+Dorsomorphin); 1 µM SB431542 (+SB431524); or 2 µM Dorsormorphin + 1 µM SB431542 (+Dual). The micropatterned H1 hESC colonies were subjected to treatment of inhibitors at either 10 hours or 34 hours after induction. Data are average of ± s.e.m of at least 6 colonies from 2 individual experiments. Asterisks indicate statistical significance (One-way ANOVA followed by Tukey´s post-test, **p<0.0001). Scale bars in A = 100 µm.*

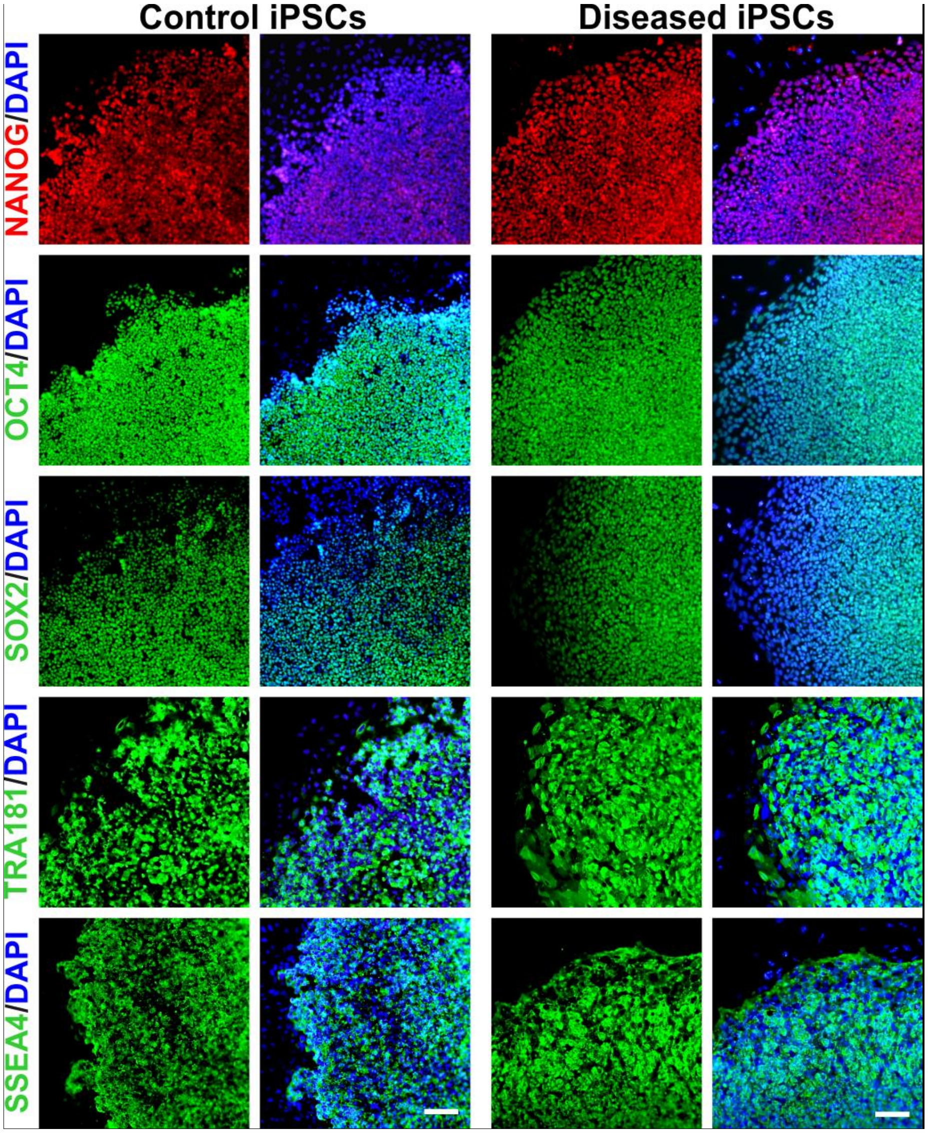

SI Figure 6. *Pluripotency characterization of control and anencephalic fetus (diseased) derived iPSCs. Positive expression of pluripotency markers, NANOG, OCT4, SOX2, TRA181 and SSEA4, indicated successful reprogramming primary dermal fibroblasts generated from skin biopsies of anencephalic fetus (diseased) and a healthy ethnically matched child (control) iPSCs. Scale bars = 100 μm.*

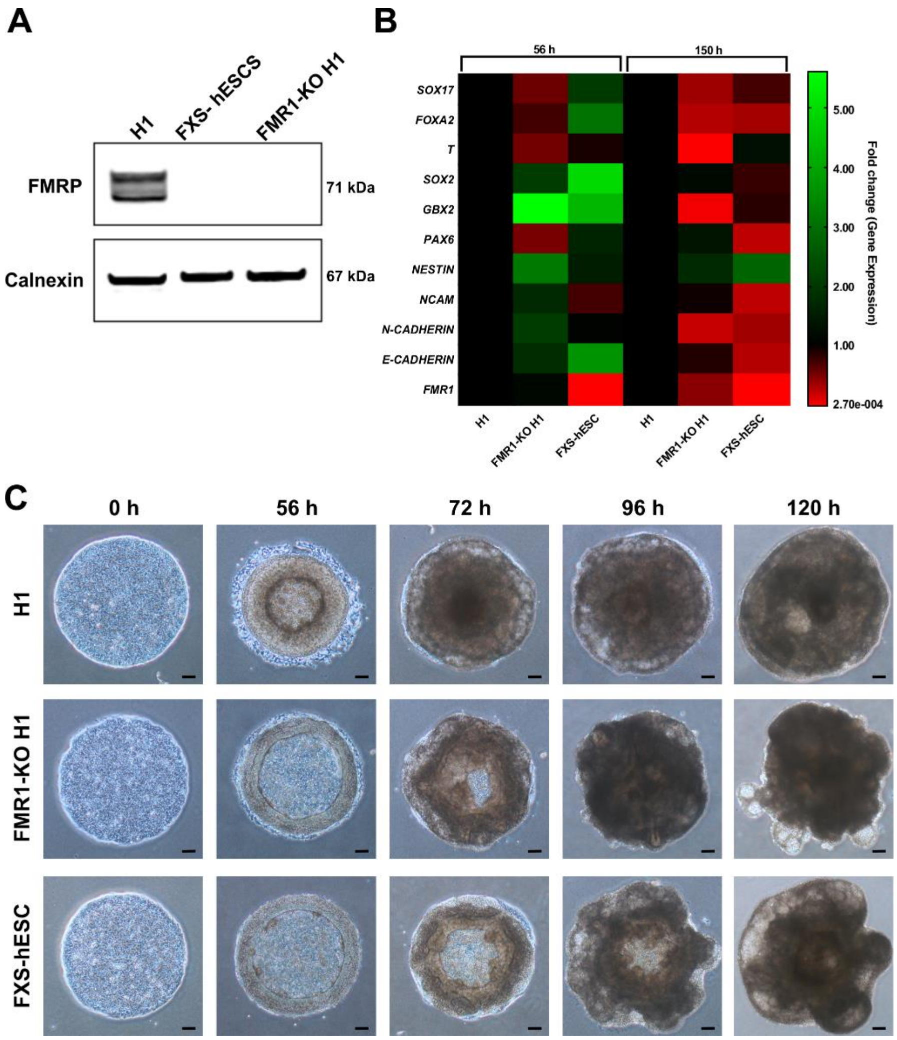

SI Figure. 7. *Molecular and structural characterization of ME-primed μNETs generated from normal H1 human embryonic stem cell (H1), an isogenic FMR1 knock-out H1 cell line (FMR1-KO H1) and a FXS human embryonic stem cell line (FXS-hESC). (A) Western blot analysis showing complete absence of FMRP proteins in FMR1-KO H1 and FXS-hESCs. (B) Transcriptional profiling of ME-primed μNETs generated from H1, FMR1-KO H1 and FXS-hESC at 56 and 150 hours after differentiation. Transcript levels are normalized to that of H1 μNETs at corresponding time points. Data are average of 3 independent experiments. (C) Gross structural morphologies of ME-primed μNETs generated using H1, FMR1-KO H1 and FXS-hESC over a time scale from 0 to 120 hours post differentiation. Scale bars = 100 μm.*

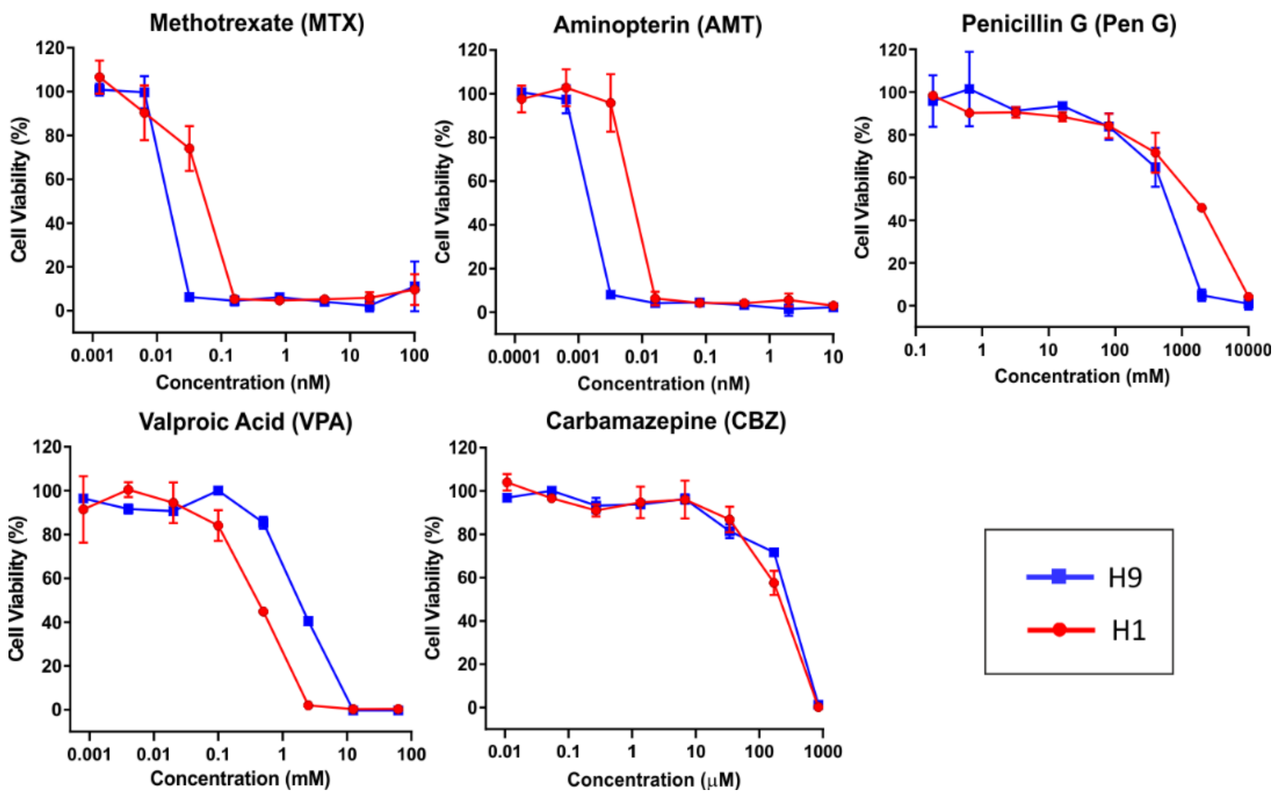

SI Figure 8. *Cell viability curves for drug treatment in human embryonic stem cells lines, H9 (blue line) and H1 (red lines) with four NTD-inducing drugs, two specific FAA (methotrexate, MTX and aminopterin, AMT) and two non-specific FAAs (valproic acid, VPA and carbamezapine, CBZ) and a non-NTD inducing control drug, penicillin G. (n = 2)*

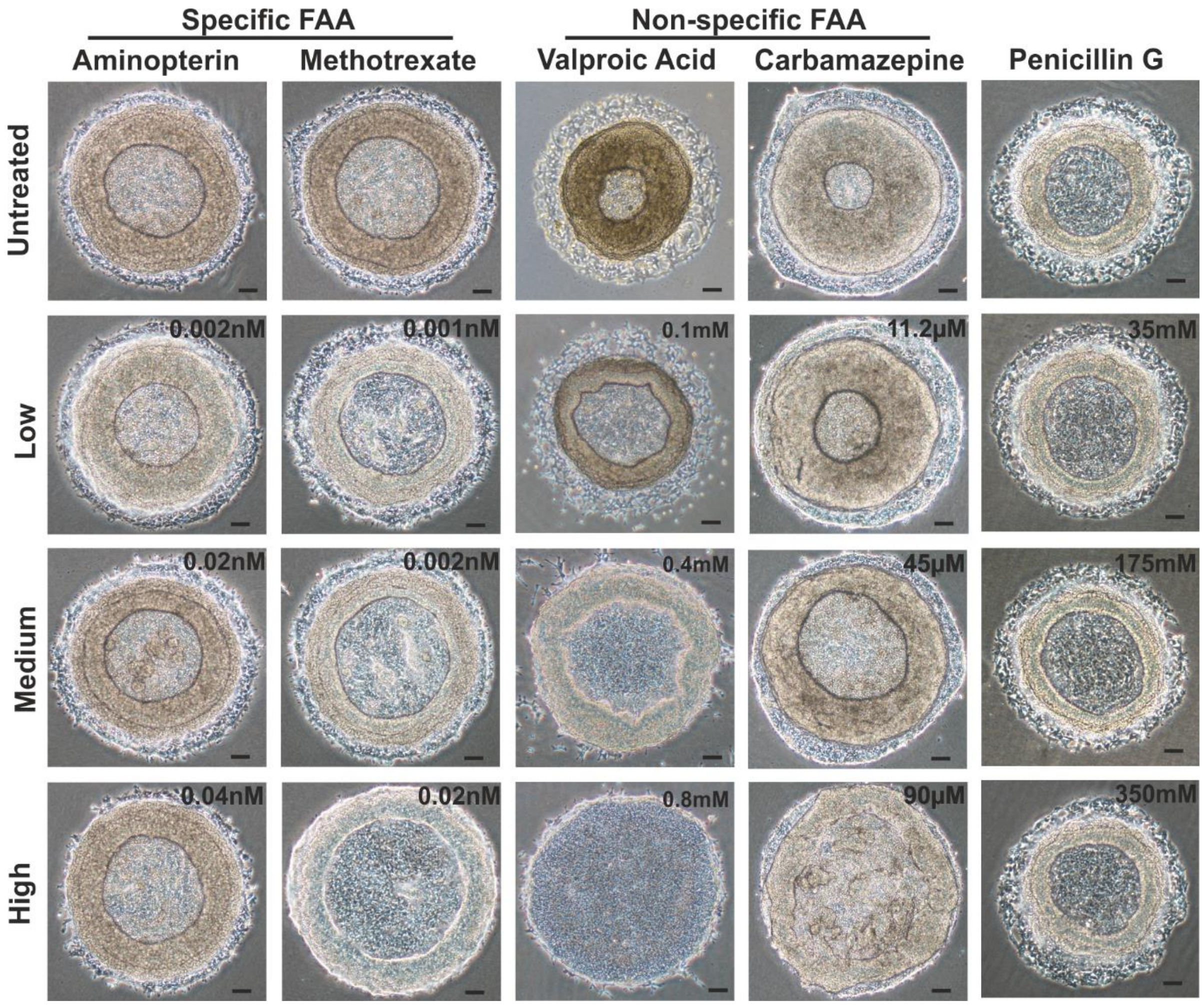

SI Figure 9. *Morphological disruption of μNETs in the presence of NTD-inducing drugs. Phase images showing dose dependent effect of specific FAA, methotrexate and aminopterin, non-specific FAA, valproic acid and carbamazepine, and a non- NTD-inducing control drug Penicillin G on the formation of 3D annular structure in μNET structures. Scale bar = 100μm. Following* IC_{25} *values, cell lines used for respective drugs are H1 for MTX, AMT, CBZ and PenG and H9 for VPA.*

Supplementary Video 1.
3D rendered videography of cross-sectioned side view of *ME-primed μNETs displaying localization of apical constriction markers ZO-1 (red) and ppMLC (magenta).*

Supplementary Video 2.
Live imaging of ME-Primed μNET starting at 34 hours post differentiation.

Supplementary Table 1. List of primers used for gene expression studies

**Gene**	**Forward**	**Reverse**
GAPDH	TGCACCACCAACTGCTTAGC	GGCATGGACTGTGGTCATGAG
SOX17	GGACCGCACGGAATTTGAAC	GGACACCACCGAGGAAATGG
FOXA2	GGGAGCGGTGAAGATGGA	TCATGTTGCTCACGGAGGAGTA
GSC	ACGCGGAGAAGTGGAACAAG	TGTCCGAGTCCAAATCGCT
T	TATGAGCCTCGAATCCACATAGT	CCTCGTTCTGATAAGCAGTCAC
MEOX1	TGGGAGCACTGCCAATGAG	GTCAGGTAGTTATGATGGGCAAA
MIXL1	TTTTCTCCCCTCTTCCAGGTAT	GGGCAGGCAGTTCACATCTA
SOX2	CCCACCTACAGCATGTCCTACTC	GTTACCTCTTCCTCCCACTCCA
PAX6	TCTTTGCTTGGGAAATCCG	CTGCCCGTTCAACATCCTTAG
GBX2	GTTCCACTGCAAAAAGTACCTCT	GAACCCTAAGATCGTCGTCCC
OTX2	CACTTCGGGTATGGACTTGC	CGGGTCTTGGCAAACAGTG
IRX3	CTCTCCCTGGTGGCGTTCTT	GTGTCCCTTCCTTCTCCATGTG
PAX2	TGTCAGCAAAATCCTGGGCAG	GTCGGGTTCTGTCGTTTGTATT
PAX5	ACCAGCAGGACAGGACATGG	TCCACTATCCTCTGGCGGAC
FGF8	AAAGCTCATCGTGGAGACGG	GCCCTCGTACTTGGCATTCT
HOXA2	ACAGCGAAGGGAAATGTAAAAGC	GGGCCCCAGAGACGCTAA
HOXA4	CCCACTGCCTCCTACTACGC	CATAGGGGTAGGCGGTGTCC
HOXA9	GGCATTAAACCTGAACCGCT	AACTGGAGGAGAACCACAAGC
HOXA11	CGCGAAGTGACCTTCAGAGA	AAATTGGACGAGACTGCGGG
PAX3	AGAAGCCGAACCACCTTCAC	GGGCCAGTTCCTCCCTAGTAT
PAX7	CAGTACGGCCAGAGTGAGTG	CCCTGACACCACCTTGTAGC
SIX1	AGAAGTCGAGGGGTGTCCTG	GGTCTCTTTGCCTCCGGTT
GATA2	GCAACCCCTACTATGCCAACC	CAGTGGCGTCTTGGAGAAG
DLX3	CTTACTCGCCCAAGTCGGAAT	AGTAGATCGTACGCGGCTTTC
NCAM	GGCATTTACAAGTGTGTGGTTAC	TTGGCGCATTCTTGAACATGA
N-CADHERIN	CCACCTTAAAATCTGCAGGC	GTGCATGAAGGACAGCCTCT
E-CADHERIN	CGAGAGCTACACGTTCACGG	GGGTGTCGAGGGAAAAATAGG
NANOG	GATTTGTGGGCCTGAAGAAA	CTTTGGGACTGGTGGAAGAA
OCT4	GGAGAAGCTGGAGCAAAACC	TGGCTGAATACCTTCCCAAA

SIP1	CAAGAGGCGCAAACAAGCC	GGTTGGCAATACCGTCATCC
NESTIN	ACAAAGTCCCTGGCCCTCTA	CACTCCCCCATTCACATGCT
FMR1	CAGGGCTGAAGAGAAGATGG	ACAGGAGGTGGGAATCTGA

Supplementary Table 2. IC_{25} values and test concentrations of drugs

**Drugs**		**IC_{25} (H1)**	**IC_{25} (H9)**
Specific Folic Acid Antagonists	Methotrexate (MTX) (nM)	0.035	0.011
	Aminopterin (AMT) (nM)	0.015	0.0011
Non-Specific Folic Acid Antagonist	Valproic acid (VPA) (mM)	0.221	0.861
	Carbamazepine (CBZ0 (µM)	85.17	92
Non-NTD inducing control drug	Penicillin G (PenG) (mM)	816.35	226.92
